# Advanced Neuroimaging of Cerebral Small Vessel Disease

**DOI:** 10.1007/s11936-017-0555-1

**Published:** 2017-06-15

**Authors:** Gordon W. Blair, Maria Valdez Hernandez, Michael J. Thrippleton, Fergus N. Doubal, Joanna M. Wardlaw

**Affiliations:** Brain Research Imaging Centres, Centre for Clinical Brain Sciences, University of Edinburgh, 49 Little France Crescent, Chancellor’s Building, Edinburgh, EH16 4SB UK

**Keywords:** Cerebrovascular disease, Small vessel disease, Magnetic resonance imaging, White matter hyperintensities, Lacunes, Microbleeds

## Abstract

Cerebral small vessel disease (SVD) is characterised by damage to deep grey and white matter structures of the brain and is responsible for a diverse range of clinical problems that include stroke and dementia. In this review, we describe advances in neuroimaging published since January 2015, mainly with magnetic resonance imaging (MRI), that, in general, are improving quantification, observation and investigation of SVD focussing on three areas: quantifying the total SVD burden, imaging brain microstructural integrity and imaging vascular malfunction. Methods to capture ‘whole brain SVD burden’ across the spectrum of SVD imaging changes will be useful for patient stratification in clinical trials, an approach that we are already testing. More sophisticated imaging measures of SVD microstructural damage are allowing the disease to be studied at earlier stages, will help identify specific factors that are important in development of overt SVD imaging features and in understanding why specific clinical consequences may occur. Imaging vascular function will help establish the precise blood vessel and blood flow alterations at early disease stages and, together with microstructural integrity measures, may provide important surrogate endpoints in clinical trials testing new interventions. Better knowledge of SVD pathophysiology will help identify new treatment targets, improve patient stratification and may in future increase efficiency of clinical trials through smaller sample sizes or shorter follow-up periods. However, most of these methods are not yet sufficiently mature to use with confidence in clinical trials, although rapid advances in the field suggest that reliable quantification of SVD lesion burden, tissue microstructural integrity and vascular dysfunction are imminent.

**Electronic supplementary material:**

The online version of this article (doi:10.1007/s11936-017-0555-1) contains supplementary material, which is available to authorized users.

## Introduction

Cerebral small vessel disease (SVD) is a condition resulting from damage to the cerebral microcirculation and predominantly affects the blood supply and tissue of the deep white and grey matter areas of the brain [1].

Radiologically, it is characterised by recent small subcortical infarcts that can present with stroke and other lesions that are usually clinically ‘silent’ until advanced such as white matter hyperintensities (WMH) in the periventricular and deep white and grey matter, enlarged perivascular spaces (PVS), lacunes, microbleeds and cerebral atrophy [2]. These features are present in up to 10% of patients in their seventh decade rising to above 85% of those in their ninth decade [3].

Clinical consequences of SVD are diverse. They include lacunar ischaemic stroke (around a fifth of all strokes), vascular and mixed dementias (SVD contributes to 45% of all dementias), intracerebral haemorrhages particularly in older people, gait and balance dysfunction and dysfunction of the bladder [1].

SVD is seen routinely on CT and MR imaging of the brain. However, structural MRI including FLAIR, T2-weighted, T1-weighted and gradient echo/T2*/susceptibility-weighted sequences (and diffusion imaging for acute lesions) are best able to characterise the full spectrum of features [2]. Increasingly, there is a desire to investigate beyond basic structural imaging in SVD as these changes may not fully explain the variance in SVD [4] and tissue changes are detectable prior to development of overt disease on structural imaging. This stems from a revised understanding of the potential pathophysiological processes and a need to detect the earliest features of SVD [1].

SVD is now understood to be distinct from large vessel atherosclerotic vascular disease [1]. Both share common risk factors such as age, hypertension, hypercholesterolaemia, diabetes and smoking [5]. However, large artery atheroma and ischaemic heart disease correlate poorly with SVD burden once a shared co-association with vascular risk factors is accounted for [6]. Instead sporadic SVD increasingly appears to be an intrinsic disorder of the endothelium that leads to lipohyalinosis, vessel wall thickening of small cerebral arterioles, fibrinoid necrosis and perivascular tissue injury [1]. There is also evidence of primary abnormalities of the matrix proteins and direct effects on myelination through impaired maturation of oligodendrocyte precursor cells, rather than just direct damage to myelin. Each potential abnormality offers possible opportunities for new interventions, hence the importance of understanding SVD pathophysiology in patients. Techniques are now developing to image endothelial and blood vessel function, in vivo, in humans, supporting the hypothesis that SVD is an endothelial disease and thus likely to require different treatments than thrombus and plaque-targeted traditional vascular therapies [7].

Imaging endothelial function whilst measuring the integrity of cerebral microstructures will increase pathophysiological understanding of SVD. The recent explosion in knowledge of SVD genetics [8] provides further insights into disease triggers and accelerators. Associating clinical consequences with specific stages, radiological features and microstructural disruption will allow targeting of therapies to the most important steps in SVD development. Of particular importance, advanced imaging methods, if valid, reliable, reproducible and relevant, can provide intermediary outcome markers of early efficacy of new interventions in patients, helping to narrow the field of potential therapies ahead of the large clinical trials that will be required to show clinical benefit by preventing stroke, functional dependence and cognitive decline [9, 10].

In this article, we will summarise recent advances in imaging SVD in the past 2 years. These have mainly occurred in three key areas: (1) quantifying SVD burden, (2) imaging microstructural integrity and (3) assessing vascular malfunction. We have not included microinfarcts, since there is a very recent update on them in submission.

## Methods for this review

We searched EMBASE, MEDLINE and PUBMED for relevant systematic reviews and primary articles between 1 January 2015 and March 2017. A separate search was performed for each section and titles were screened by GB or MVH (see supplementary information). Additionally, we hand-searched the *Journal of Cerebral Blood Flow and Metabolism* over the same period, checked references in opinion reviews and utilised the authors personal libraries of relevant articles in neuroimaging and SVD.

We used recent systematic reviews where available as these are generally the best summaries of the available evidence, updated with recent publications. We assessed publications for study size, patient characteristics, imaging methods and feature sought, evidence of validation, of adjustment for co-related variables in pathophysiological analyses and extracted information on the main results. We did not use a quality assessment tool as this work was not a formal systematic review and the literature was very diverse.

### Quantification of SVD features

**Fig. 1:**
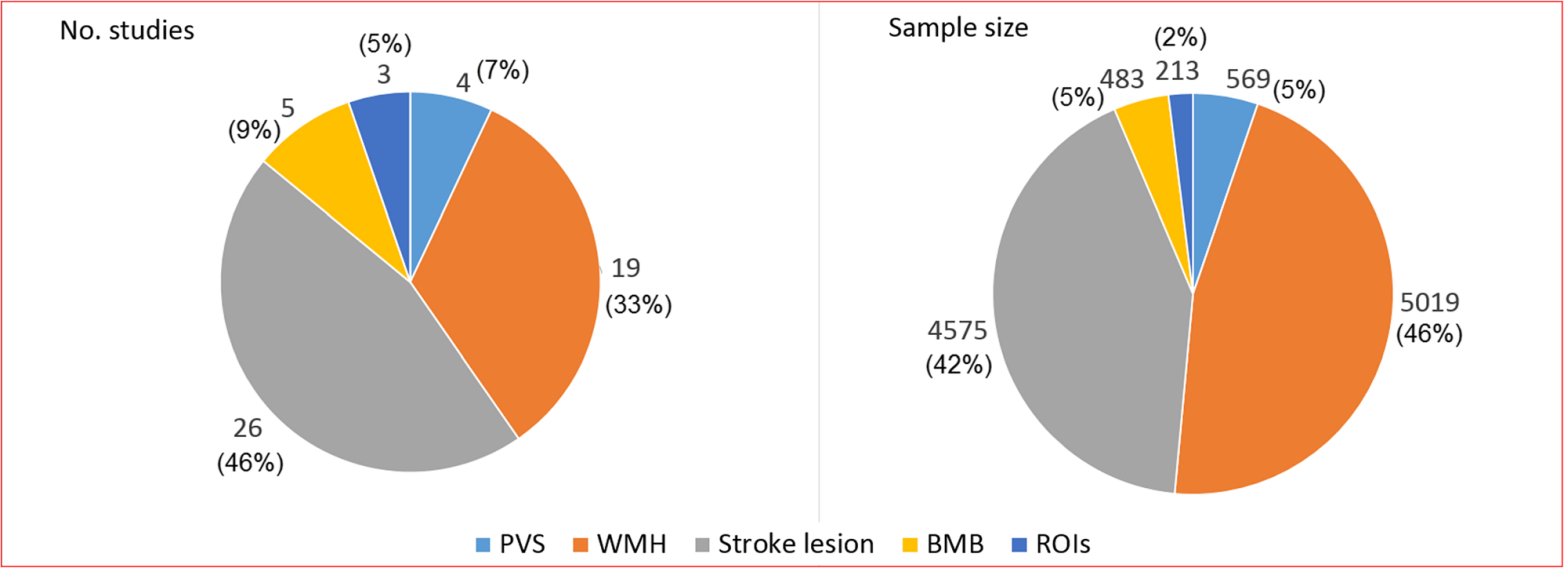
Graphical representation of the proportions of papers published since January 2015 until March 2017 on computational methods to assess small vessel disease imaging markers and sample size involved. Legend: *PVS* perivascular spaces, *WMH* white matter hyperintensities, *BMB* brain microbleeds, *ROIs* regions of interest.

In recent years, efforts have been made to quantitatively assess specific SVD markers on one hand and to devise metrics that allow the quantification of the total burden of SVD on the other. We identified 48 articles since the start of 2015 that have attempted to computationally assess either WMH, ischaemic stroke lesions, PVS, cerebral microbleeds or regions of interest reportedly related to vascular dementia and SVD in general (Fig. 1 and Supplementary Table S1A). In the same period, we identified nine articles that used a composite score reflective of the total SVD burden (Table 1).

**Table 1:**
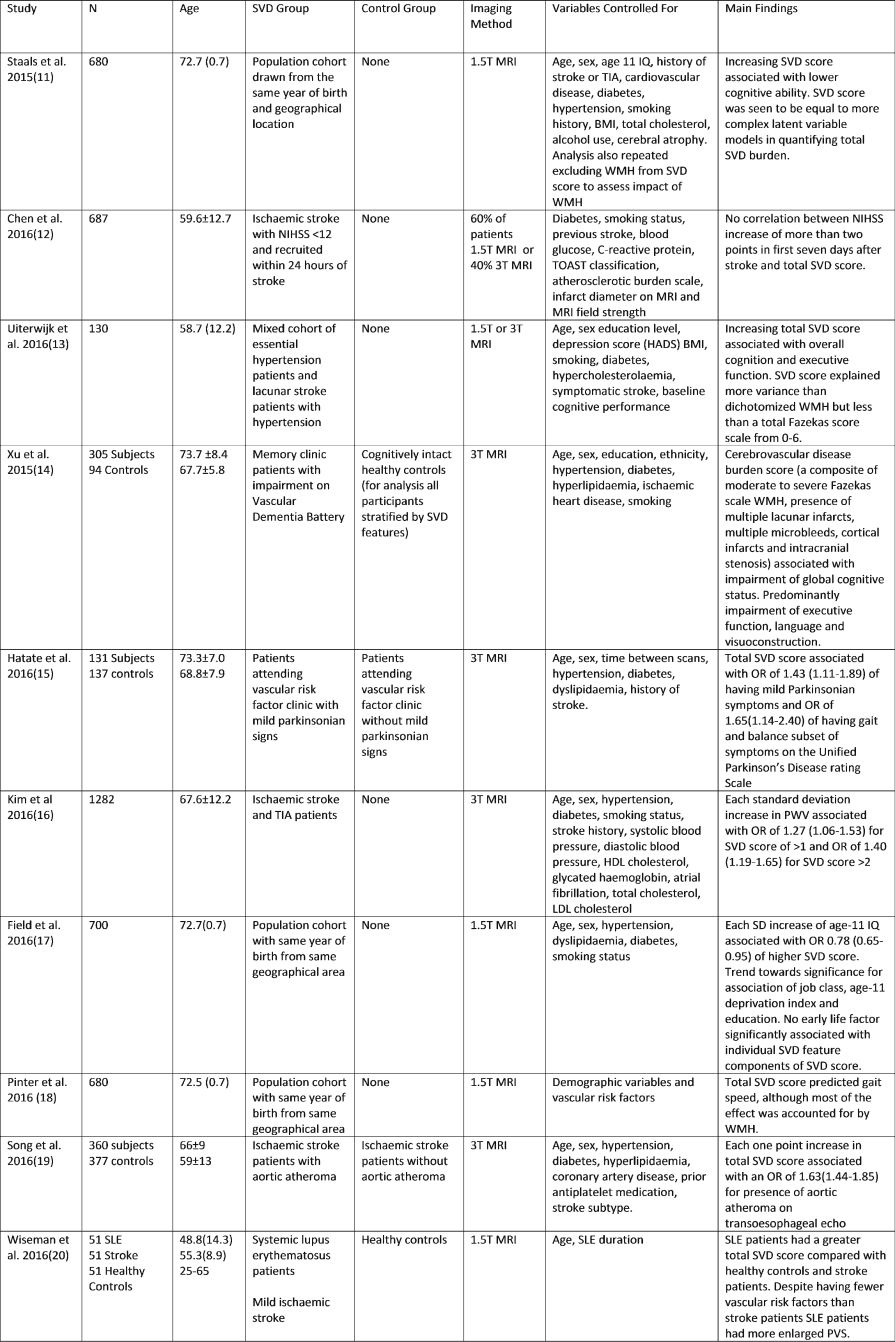
Original papers published since 1 January 2015 on composite SVD scores

Classical machine learning-based classification algorithms like support vector machines (SVMs) and random forest lead the list of computational methods applied to the assessment of SVD markers (16/48 studies in this period). They have shown promise for making high dimensional neuroimaging data clinically useful, but identifying imaging-based patterns that contribute significantly to classifier decisions remains an open problem. This is an issue of critical importance in imaging studies seeking to determine which anatomical, textural or physiological imaging features contribute to the classifier’s decision, thereby allowing users to evaluate critically the findings of such machine learning methods and to understand disease mechanisms. The majority of published work addresses the question of statistical inference for support vector classification using permutation tests based on SVM weight vectors. Such permutation testing ignores the SVM margin, which is critical in SVM theory. A study proposes a metric attempting to account for this margin [21], but given the complexity of the global feature space in which coexist several manifolds, the approach of separating ‘classes’ using a hyperplane does not always have the best outcome. Deep learning approaches like convolutional neural networks offer a solution to this problem. And, indeed, several studies have applied them to assess SVD markers with promising results [22–26]. But still, conventional thresholding and clustering approaches are being redefined in novel ways to address the challenging issue of coping with the wide variation of abnormalities present in the brain images of individuals with SVD. However, the application of these recently developed methods to clinical research is still premature. Some of them have been developed using only a few representative datasets. Collaborative efforts that allow them to be evaluated in large multicentre datasets and in samples representative of the whole SVD spectrum are needed.

From the attempts to devise a composite SVD score representative of the individual’s SVD burden, a major effort has been made by Staals and colleagues [27•], based on visually rated individual SVD features and combines them into the ‘total SVD burden’ on a scale of 0–4 [27•]. Initial efforts were further tested and refined in larger datasets of patients with stroke and healthy older individuals, testing inclusion of additional features and different weighting of individual feature scores, with the Edinburgh group [11, 17, 18]. In the current version of the score, a point is attributed for each of the following: moderate to severe WMH, presence of moderate to severe PVS in the basal ganglia, presence of lacunes and presence of microbleeds.

This SVD score has been reported by multiple research groups to be associated to cognitive function. Staals et al. found in 680 community-dwelling healthy elderly subjects of the Lothian Birth Cohort 1936 (LBC1936) that a higher SVD score was associated with lower cognitive ability in older age and performed well against an ‘SVD burden’ constructed using latent variable modelling [11]. Uiterwijk et al. found an association of the SVD score with cognitive decline over 4 years and specifically in the domains of executive function and processing speed, with higher scores at baseline in patients with hypertension [13]. Other studies have found the total SVD score was also associated with gait and balance impairment in healthy older subjects [18] and patients with vascular risk factors [15]. Using a different composite score that looked at a broader range of cerebrovascular disease burden incorporating both small and large vessel contributions, Xu et al. also showed an association of their composite CeVD score with reduced global cognitive function and particularly impairment of executive function, language and visuoconstruction [14].

Total SVD burden measures have also been associated with emerging SVD risk factors. These include early life factors such as age 11 IQ, childhood deprivation, education and class of occupation in the LBC1936 [17]. Pulse wave velocity (PWV, a marker of arterial stiffness) which has previously been repeatedly shown to correlate with WMH has shown similar associations with a composite SVD score [16]. One study has also linked aortic plaque to total SVD severity [19]. Composite SVD scores have been used in investigations of other medical conditions that may influence brain health, e.g. mild parkinsonian signs [15] and in systemic lupus erythematosus [20]. One study also looked to see if total SVD score could predict early neurological deterioration after stroke but found no association [12].

A recent systematic review has highlighted the reproducibility and variability in measuring SVD imaging features computationally and the factors that affect these [28••]. The most studied features were brain atrophy (19 articles), WMH (five articles) and microbleeds (four studies) with no studies addressing reproducibility of PVS or lacunes. Whilst good within-centre reproducibility was seen for brain atrophy and WMH, this was in small studies and between-centre variability was much higher. Field strength, sequence type, scanner manufacturer, head coil, patient positioning and impact of scanner upgrades could all affect reproducibility and variability [28••]. Larger studies of between-centre reproducibility are required and study protocols and analysis should account for these factors.

### Measuring structural integrity

White matter structural and functional integrity can now be investigated in greater detail with diffusor tensor imaging (DTI) techniques. The most common derived measures are fractional anisotropy (FA) and mean diffusivity (MD). FA decreases and MD increases as white matter is damaged and diffusion is not restricted to a single direction along the paths’ of axons. These provide a quantitative measure of integrity in a given tract or region. Emerging techniques are allowing these metrics to be converted into maps of connectivity or ‘connectome’. We identified 26 articles (*n* = 17,188) investigating SVD and white matter integrity measured with DTI (Table S2).

A recent systematic review found DTI had been used to investigate white matter integrity and SVD risk factors in one study of 499 patients (published in four journal articles); cognition in SVD in 19 articles (*n* = 3620 patients, although the number falls to 1402 patients when multiple articles on the same cohorts are accounted for); depression in SVD in four studies (*n* = 733 patients); motor effects of SVD in eight studies (*n* = 592 patients); and cerebral autosomal dominant arteriopathy with subcortical infarcts and leukoencephalopathy (CADASIL) in six studies (*n* = 307 patients). In general, the review found that FA was decreased and MD increased in WMH and normal appearing white matter (NAWM). NAWM was more abnormal close to WMH. Evidence that risk factors were associated with microstructural damage in SVD was relatively sparse and came from a single cohort (*n* = 499) but suggested that hypertension, smoking and physical inactivity increased microstructural damage in NAWM although some of these associations disappeared after correction for other SVD features. Cognition remained associated with white matter integrity after taking into account other imaging markers of SVD. Longitudinal associations between DTI metrics and declining cognition are less well established however. Whilst depression has been linked with microstructural integrity, this relationship disappeared when other SVD imaging markers or disability were controlled for. Motor symptoms of SVD have been more consistently linked to NAWM integrity, specifically disturbances of frontal microstructure, although this was based on studies covering only five cohorts (*n* = 681). Overall, this review concluded that DTI indices were associated with clinical features of SVD and that many of these relationships remained apparent after correcting for other imaging markers of SVD [29].

A further recent systematic review assessed the relationship of diabetes to SVD [30]. Five of the 49 studies (*n* = 590) included in the review used DTI measures and all had shown that FA was decreased and MD increased in patients with diabetes [30].

Other studies published since these reviews found similar results but emphasised that factors such as demographic variables and other SVD imaging features must be accounted for when utilising DTI metrics to avoid confounding [31–43].

The prognostic significance of decreased white matter integrity was not assessed in these systematic reviews but some data have been published recently. In 4259 subjects from the Rotterdam Study cohort, who were stroke-free at inception, with over 18,476 person years of follow-up, impaired global white matter integrity on DTI was associated with increased stroke risk: FA, hazard ratio for stroke was 0.75 (0.57–0.98) per standard deviation increase in FA; MD, HR 1.50 (1.08–2.09) per standard deviation increase in MD. The effect was independent of age, sex, cardiovascular risk factors, WMH volume and lacunes [44]. Each standard deviation decrease in FA and each SD increase in MD were also associated with an increase in all cause mortality (HR 1.37 (1.20–1.57) and 1.49 (1.28–1.75), respectively) over 5.4 years of follow-up, controlled for demographic variables, vascular risk factors and other SVD imaging markers. Further analysis indicated that the increased risk of death with falling FA was specifically for cardiovascular not non-cardiovascular death [45].

Methods to increase the sensitivity of DTI measures to early white matter injury are now being developed, for example the metric of ‘peak width of skeletonized mean diffusivity’ (PSMD) [46]. This method skeletonises the white matter tracts reduced the contamination from CSF containing structures and increased sensitivity to the whole brain burden of SVD. PSMD was associated with slower processing speed in patients with CADASIL and in patients with sporadic SVD (but not in healthy controls or in patients with Alzheimer’s Disease) and suggested that PSMD was more sensitive to progression of brain injury from SVD than the individual features of WMH volume, lacune volume or brain volume. Estimated sample sizes for a clinical trial with 18-month follow-up suggested that PSMD had the smallest required sample size compared to whole brain MD peak height, normalised WMH volume, brain parenchymal fraction, processing speed score or normalised lacune volume [46]. Other studies have assessed the use of DTI measures in clinical trials. In SCANS (patients with symptomatic lacunar infarcts and confluent WMH), changes in MD, brain volume, WMH volume and lacunes were detectable over 3 years of follow-up but not in cognitive scores: the lowest estimated sample size was from WMH volume, followed by MD in NAWM [9]. This requires further testing in other cohorts, since an analysis of 1-year follow-up in about 200 patients with minor stroke and WMH in the Mild Stroke Study 2 suggested that failure to account for WMH reduction as well as increase resulted in huge sample sizes [47].

#### Connectivity imaging

The emerging field of connectomics allows DTI metrics to analyse the strength and complexity of white matter connections between different brain regions and the efficiency of these networks. For example, *nodal efficiency* is a measure of the path length between a node and all the other nodes in a network. Shorter path lengths are more efficient. A decrease in nodal efficiency means communications are being routed by a longer path.

In a cohort of 232 patients with cognitive impairment of mixed aetiology, WMH volume and lacunes were associated with decreased nodal efficiency in frontal, lateral temporal, lateral parietal and occipital regions. Using path analysis, frontal nodal efficiency appeared to mediate the effects of SVD on frontal atrophy and frontal executive dysfunction. Temporal and parietal nodal efficiency appeared to mediate the effects of SVD on temporal and parietal atrophy and memory dysfunction [32].

In 38 patients without dementia but with probable CAA (based on Pittsburgh B PET imaging), including 17 participants with intracerebral haemorrhage, lower global network efficiency was related to higher cortical amyloid load, WMH volume, atrophy and a trend towards significance for cerebral microbleeds. Lower network efficiency was also related to worse performance on processing speed and executive function tests and gait velocity but not memory. The association of network efficiency with cognition persisted after controlling for the SVD, but it was not stated how it compared to a simple FA value [48]. A similar association with cognition was found in 27 patients with a lacune on neuroimaging where global and local network efficiencies were reduced compared to control and memory, attention, executive function and verbal fluency were all associated with nodal efficiency [49].

Connectivity can also be investigated using resting state BOLD MRI techniques to identify networks. In 19 patients with symptomatic subcortical stroke producing motor deficits, functional connectivity assessed with resting state BOLD MRI, between the M1 region and motor cortex in the contralateral hemisphere, was reduced post-stroke. Structural connectivity in the corpus callosum measured using fibre tracking was also reduced compared to controls [50].

Although promising as sensitive markers of early SVD-related brain damage, considerably more work in different populations and particularly in longitudinal studies is required to establish if structural or functional connectivity can provide accessible, reliable, reproducible or relevant measures of microstructural damage or could be utilised as early efficacy markers in clinical trials.

### Imaging microvascular malfunction

Several non-invasive techniques are available to investigate cerebral haemodynamics in vivo. At the simplest, these estimate cerebral blood flow but can be expanded to measure pulsatility throughout the cardiac cycle, blood flow reactivity, blood-brain barrier leakage and metrics related to oxygen utilisation. We identified 22 articles relevant to SVD using these techniques (Table S3).

#### Cerebral blood flow and arterial stiffness

A recent systematic review and meta-analysis summarised current literature to the end of 2015 on cerebral blood flow (CBF) and the most commonly studied SVD feature, WMH. Four studies assessed CBF and WMH longitudinally whilst 34 were cross-sectional, 24 of which were suitable for meta-analysis. In cross-sectional analysis, CBF (mostly measured in cortex or whole brain) was lower in subjects with more WMH [51••], but this relationship was attenuated when subjects with dementia and non-age-matched studies were excluded [51••]. Few studies measured CBF in either NAWM or in WMH. The four longitudinal studies gave conflicting evidence: the largest (*n* = 575 patients [52]) found that high WMH burden predicted falling CBF at follow-up but low CBF did not predict increasing WMH in the long term; in the next largest (*n* = 390), low CBF predicted progression of periventricular WMH only [53]); in 74 subjects, CBF increased in some regions with WMH progression [54]; and the smallest study (*n* = 40) found low CBF in regions where WMH developed at follow-up [55].

Peripheral arterial stiffness and its relationship to SVD imaging features (including WMH, microbleeds, lacunes) and cognition were addressed in a systematic review that included 23 studies (*n* = 15,666 participants) [56]. Eleven of 15 studies of PWV or carotid stiffness and 6/12 studies of pulse pressure showed an association between increasing arterial stiffness and SVD features. A pooled analysis was possible for studies using PWV measures, showing that a one standard deviation increase in PWV was associated with an increase in SVD features (ORs between 1.29 and 1.32, *p* < 0.001) [56]. Sensitivity analysis showed little effect of excluding studies considered to be at high risk of bias. There were 41 papers (*n* = 57,671 subjects) on the association between PWV and cognition but between study heterogeneity precluded any pooled analysis. In terms of individual studies of PWV, 6/11 cross-sectional studies found an association global cognitive impairment, 5/22 cross-sectional studies found an association with individual cognitive domains and 3/4 longitudinal studies found an association with global cognitive decline. Pulse pressure was associated with global cognitive impairment in 3/13 longitudinal and 4/13 cross-sectional studies [56].

Table S3 lists studies published since January 2015, most of which were small (median *n* = 106) and found similar associations between PWV or pulse pressure and SVD lesions or cognition [57–61]. The most relevant recent data come from the Discontinuation of ANti-hypertensive Treatment in the Elderly (DANTE) study [62]. DANTE provided a cross-sectional analysis of CBF and blood pressure in 203 stroke-free, community-dwelling participants with some cognitive impairment (MMSE 21–27) and a longitudinal sub-analysis of 102/203 participants randomised to discontinue versus continue anti-hypertensive treatment. Cross-sectionally, there was no association between CBF and any of systolic BP, diastolic BP, pulse pressure, mean arterial pressure or change on standing of systolic or diastolic BP in an age- and sex-adjusted analysis, regardless of WMH volume, presence of microbleeds or lacunes. Longitudinally, the change in CBF over 4 months follow-up did not differ between those who continued versus those who stopped anti-hypertensive therapy. These findings from an interventional trial suggest that blood pressure is not a major influence on CBF in older cognitively impaired subjects despite hypertension being one of the strongest risk factors for SVD. This further highlights that reduced CBF does not fully explain the development of SVD.

A modest literature on intracranial vascular stiffness, mostly assessed using transcranial Doppler (TCD), was summarised in a systematic review prior to 2015 [63]. Intracranial arterial flow velocity, assessed with TCD, is well established to increase with age and WMH score independent of vascular risk factors (age = 0.2 cm s^−1^ fall in velocity/year increase in age, *p* = 0.045; WMH score, 3.75 cm s^−1^ fall in flow velocity/point Fazekas score increase, *p* = 0.004) [64] and others have suggested that middle cerebral artery velocities assessed with TCD could be used as a proxy measure of WMH burden [65]. However, the relationship between flow velocity, CBF and pulsatility is complex. Some researchers are now starting to investigate other flow parameters including transit times and intracranial blood flow pulsatility. Transit times may reflect compensatory increase in blood volume to counter reduced CBF or slower transit due to longer vascular pathways for example due to shunting or otherwise dysregulated flow [66]. Consistent with the latter theory, Arba and colleagues examined CBF, cerebral blood volume (CBV) and MTT in 120 patients with acute stroke and found a near-linear increase in transit time with increasing WMH score [67], with much less effect on CBF or CBV. The falling flow velocity may reflect increasing pulsatility, since the two tend to go hand in hand; however, whilst intracranial measures of arterial stiffness are clearly related to increasing age [68, 69], evidence of a direct relationship to SVD features is currently weak [69, 70]. Notwithstanding the clear associations between systemic arterial stiffness and cerebral SVD imaging features, an association with intracranial stiffness should not be assumed and studies with better defined SVD populations corrected for demographics and risk factors are required. In the future, it is now possible to image pulsatility and blood flow velocities in individual perforating arterioles down to 80 μm in diameter with 7 T MRI [71•].

#### Cerebrovascular reactivity

Dynamic function of the cerebral blood vessels at voxel level is now an accessible technique. Cerebrovascular reactivity (CVR) measures the brain’s vascular response to a vasoactive stimulus, such as inhaling carbon dioxide (CO_2_). Combining hypercapnia with MRI allows tissue level quantification of CVR and is therefore an advance on cerebral vasomotor response assessed with TCD of the middle cerebral artery [63].

We systematically reviewed all studies of CVR in SVD between 1990 and 2015 and found five studies (*n* = 155), but these provided insufficient data to determine the relationship between CVR and SVD [72]. Two of five relevant studies (*n* = 17) showed that decreased CVR was associated with increased WMH but included only 11% of the patients in the five studies. The other three larger studies showed no association. CVR was also shown to decrease with age in three studies and female gender and diastolic BP in one study [72]. Clearly more data are needed.

Since this review, CVR has been assessed in two published studies. CVR was lower in areas of NAWM that became WMH in a longitudinal study (*n* = 45) [73]. Areas of negative CVR, i.e. reduced flow during CO_2_ inhalation suggesting areas are at risk of ischaemia due to vascular steal, have also been associated with lower CBF and microstructural damage in a study of 75 patients [74]. Whilst intriguing, further studies controlling for age, vascular risk factors and baseline WMH volume are needed.

We assessed CO_2_-challenge MRI CVR (and in parallel assessed intracranial vascular pulsatility) in an observational cross-sectional study (*n* = 60) [75] in which we found associations between falling CVR and increasing WMH burden and increased PVS visibility (and increased pulsatility and WMH burden). We and others are now determining if the CO_2_ MRI CVR method can be used in multicentre observational studies (INVESTIGATE@SVDs ISRCTN10514229) and in randomised clinical trials testing potential SVD treatments as an intermediary marker of efficacy (LACI-1 ISRCTN 12580546; TREAT@SVDs Clinicaltrials.gov registration in process). Determining if the CVR method is sensitive and reliable enough to use in multicentre studies would make a potentially very powerful technique available for large-scale trials and population studies.

#### Blood-Brain barrier (BBB) leakage

The blood-brain barrier (BBB) normally regulates material entering and leaving the brain to maintain homeostasis of the brain cell environment. However, it becomes subtly more leaky with advancing age [76], very obviously more leaky following acute ischaemic, traumatic or oncological injury, and it is subtly more leaky than normal in SVD. Measures of BBB permeability in vivo are available to assess cerebral vascular endothelial integrity such as dynamic contrast enhanced MRI and CT perfusion techniques although caution is required since methods to assess subtle BBB leak are still evolving.

Until February 2014, 70 studies had been performed using DCE-MRI to assess BBB permeability that covered 417 individual animals and 1564 humans. Of conditions potentially relevant to SVD, BBB leakage was assessed in 482 stroke patients, 30 Alzheimer’s disease (AD) patients, 22 mild cognitive impairment (MCI) patients and 20 diabetic patients. Differences in diseased subjects compared to controls were found in acute ischaemic stroke, vascular cognitive impairment, mild cognitive impairment, SVD and AD. Two studies were identified that reported impaired BBB permeability in lacunar versus cortical stroke.

A recent systematic review on BBB leakage in humans during ageing, in SVD or dementia, identified six studies including 203 patients [77]. This identified that BBB was more permeable in white matter and deep grey matter in those with diabetes-associated MCI, more permeable in lacunar stroke patients than atherothromboembolic stroke in white matter and CSF and more permeable in white matter in patients with WMH and both AD and vascular dementia and in the hippocampus in patients with MCI.

We assessed BBB leakage in 204 patients with minor stroke, doubling the sample available in the literature. As well as differences in WM integrity between WMH and NAWM, permeability assessed by DCE-MRI slope enhancement was increased in WMH compared to NAWM [43, 77, 78], and in NAWM close to versus remote from WMH, when adjusted for age, WMH burden, vascular risk factors and important methodological variables such as baseline T1 value and time after contrast injection. Additionally, leakage increased with age, hypertension and WMH severity and predicted declining cognition at 1-year follow-up [77].

Other small studies in the last few years have found similar results [79–81], although many did not adjust for important demographic variables and risk factors [79, 80] (Table S3).

Despite the growing interest in assessing low-level BBB leakage using DCE-MRI, it should be noted that this is a very difficult technique with imaging, since the signal changes caused by slow leakage are small, take a long time to acquire and are typically of comparable magnitude to the level of noise, artefact and instrumental or biological signal drift all of which have to be accounted for. Most prior studies have not done this. There is also no agreed minimum standard acquisition protocol, signal modelling method and parameters to be reported. Several groups have reported on the pitfalls and limitations of the technique, and the results of which should be interpreted with caution [78, 82–84]. The standardisation of the BBB leakage method for studies in SVD is now being assessed in an international Working Group (*HARNESS*) funded by the multinational Joint Programme for Neurodegenerative Diseases (JPND).

## Conclusion

SVD remains a condition of many unknowns. Known SVD vascular risk factors do not fully explain the variance in SVD development. Clinical consequences are diverse: some individuals are symptomatic at early stages, and others have large radiological disease burdens and no apparent symptoms. SVD even appears reversible in some individuals. There remains no effective, specific treatment to halt radiological or clinical progression of SVD.

The realisation that SVD is distinct from both large vessel atherosclerotic vascular disease and normal ageing has only been accepted relatively recently. The increased availability of increasingly sophisticated neuroimaging that can unpick differences in tissue structure and vascular function and relate these closely to symptoms in people has been key to this. Further development of advanced neuroimaging techniques holds the key to improving our knowledge and treatment of SVD, but crucially, this must be accompanied by demonstrating that the methods are reliable, repeatable, relevant, tolerable and accessible to affected patients.

In this review, we have described how better quantification of the total brain burden of SVD may help stratify patients more effectively than using individual features and improve the understanding of the causes and consequences of SVD. Imaging microstructural integrity and vascular malfunction are allowing us to identify much earlier disease stages and tissue at risk of progression to more established, later stage SVD brain damage. The focus of studies in SVD should not only be to limit progression of brain damage but to try to reverse it before it becomes permanent. Whilst these tissue level metrics have the potential to help understand what structures are damaged, and in what way, in each of the specific clinical consequences of SVD, most of these methods are not yet mature enough for use in large clinical trials.

Finally, an improved understanding of the pathophysiology of SVD, and its drivers at different disease stages, will identify new treatment targets. The use of more sensitive advanced imaging metrics as surrogate outcomes then also has the potential to facilitate clinical trials with smaller samples and shorter follow-up periods to accelerate discovery of effective treatments as efficiently as possible.

## Electronic Supplementary Material

Table S1 (DOC 145 kb).

Table S2 (DOC 322 kb).

Table S3 (DOC 41 kb).

ESM 1 (DOC 207 kb).
