# Supplementary material for: Advanced Neuroimaging of Cerebral Small Vessel Disease
Source: Curr Treat Options Cardiovasc Med. 2017 Jun 15;19(7):56. doi: 10.1007/s11936-017-0555-1 (PMC5486578; doi:10.1007/s11936-017-0555-1)
Supplement: Supplementary file 1 — (DOC 145 kb). [file 11936_2017_555_MOESM1_ESM.doc]

**Table S1:** Original papers published since 1^st^ January 2015 on computational image processing in SVD

| Method | Purpose | SVD marker | References | Sample Characteristics | CT/MR sequences used | Validation results |
| --- | --- | --- | --- | --- | --- | --- |
| Automatic scheme that uses scale-invariant feature transform descriptors extracted from a dense grid of patches from the ROI on a support vector machine classifier | Dichotomous classification of PVS burden | Peri-vascular spaces | [1]  10.1007/978-3-319-41501-7_72 | 264 (110 F) mild stroke patients mean age 66 years old (SD 11 years) | Routine clinical T2-weighted MRI at 1.5T | accuracy 82.3%, sensitivity 83.9%, specificity 80.8% |
| Textural characterisation of ROI analysis | Characterisation of ROIs to investigate SVD in patient groups | Stroke lesions, WMH and normal tissues | [2] | 42 mild stroke patients mean age 66 years old (SD 11 years) | Fast spin gradient echo 12o and FLAIR | Tissue charac-terisation agrees with clinical reports |
| Automatic scheme that uses discrete wavelet transform and texture from a ROI on a support vector machine classifier | Dichotomous classification of PVS burden | Peri-vascular spaces | [3]  [10.1016/j.procs.2016.07.003](http://dx.doi.org/10.1016/j.procs.2016.07.003) | 264 (110 F) mild stroke patients mean age 66 years old (SD 11 years) | Routine clinical T2-weighted MRI at 1.5T | accuracy 80.0%, sensitivity 80.7%, specificity 79.4% |
| Automatic segmentation using Frangi filter optimised using the ordered logit model and visual ratings as priors for the filter parameters | Segmentation of vessel-like structures | Peri-vascular spaces | [4]  [10.1016/j.procs.2016.07.011](http://dx.doi.org/10.1016/j.procs.2016.07.011) | 24 community-dwelling adults 72.3 (SD 0.6) years old | Routine clinical T2-weighted MRI at 1.5T | Spearman ρ with visual rating scores 0.75 (volume) and 0.69 (count) |
| Convolutional Neural Network | Classification into Atrophy, present lesion with mass effect and normal | Large cortical stroke | [5]  [10.1109/SAI.2016.7555958](https://doi.org/10.1109/SAI.2016.7555958) | 282 adults: 51 with Alzheimer’s disease, 118 with a mass lesion and 113 normal | CT scans | Accuracy 87.7% |
| Convolutional Neural Network | Segmentation of large cortical stroke in brains with none or mild WMH burden | Large cortical stroke | [6]  [10.1109/ICIP.2016.7532329](https://doi.org/10.1109/ICIP.2016.7532329) | 18 patients with aphasia due to large single left-hemisphere stroke | T1-weighted 3D isotropic MPRAGE | Mean Dice similarity coefficient 0.78 (SD 0.04) |
| Generative probabilistic model that generalizes the Gaussian mixture model followed by the expectation maximization algorithm. | Lesion segmentation | Evaluated only in cortical stroke lesions | [7]  10.1109/TMI.2015.2502596 | 18 patients with cortical strokes | FLAIR, T1-, T2-weighted (the latter before and after contrast) | Dice similarity coefficients close to 0.8 |
| Regional sparse learning and Markov random field regularisation. Image decomposition to identify deviations from a normative database. | Lesion segmentation | WMH and cortical infarcts | [8]  [10.1109/TMI.2016.2538998](https://doi.org/10.1109/TMI.2016.2538998) | 34 patients with WMH and/or cortical infarcts mean ages:  60.8 (SD 5.8) training set and 75 (SD 4.7) testing set | FLAIR. Multi-modality extension discussed but not evaluated | Box plots of AUC (0.97-0.99) and Hellinger Distance (4.5-5.1) |
| Registration of a tissue probability atlas to FLAIR following by thresholding | Lesion segmentation | WMH | [9]  [10.1145/2851613.2851938](https://doi.org/10.1145/2851613.2851938) | 10 multiple sclerosis patients | FLAIR | Mean Dice 0.5, mean Sensitivity 0.89 |
| Pipeline using Skull Elimination Algorithm (SEA), Central Line Sketching Algorithm Fuzzy C-means clustering-based segmentation and Discrete Orthonormal Stockwell Transform | Lesion segmentation | Stroke lesions | [10]  [10.1504/IJBET.2016.076731](http://dx.doi.org/10.1504/IJBET.2016.076731) | Paper not freely available and information not provided in the Abstract | | |
| CAD semi-automatic system that uses 12-feature-based voxel classification followed by an object classifier, both being random forest classifiers with 100 trees each | Segmentation | Brain Micro-bleeds | [11]  10.1016/j.nicl.2016.07.002 | 33 patients with traumatic brain injury | T1-weighted and SWI | Mean sensitivity 89.1%(SD 0.8%) |
| Automated pipeline that uses the Expectation Maximization algorithm interleaved with a modified Markov Random Field followed by the Least Absolute Shrinkage and Selection Operator | Lesion segmentation | WMH | [12]  10.1016/j.nicl.2016.05.018 | 107 children with unilateral cerebral palsy and 18 healthy children | T1-MPRAGE at 3T and T2-Turbo-FLAIR | Accuracy 93.6%, Sensitivity 93.9%, Specificity 92.9% |
| Automated tool (BIANCA) based on the k-nearest neighbour algorithm | Lesion segmentation (Brain Intensity AbNormality Classification Algorithm) | WMH and stroke lesions | [13]  [10.1016/j.neuroimage.2016.07.018](http://dx.doi.org/10.1016/j.neuroimage.2016.07.018) | 85 older adults (neurodegenerative cohort) and 474 patients with minor stroke or TIA (vascular cohort) | FLAIR at 3T | Mean Dice 0.75 and 0.52. Correlation with Fazekas 0.76 and 0.84 |
| Gamma correction extreme-level-eliminating with weighting distribution | Lesion identification (CT contrast enhancement) | Ischaemic lesions | [14]  10.1002/sca.21334 | 5 CT datasets | CT scans | Peak SNR 21-25, enhancement by entropy 2.5-4.5 |
| Extraction of features through patterns of voxels that represent lesion probability | Decode post-stroke functions from structural brain MRI | - | [15]  [10.1016/j.nicl.2016.07.014](http://dx.doi.org/10.1016/j.nicl.2016.07.014) | 50 stroke patients mean age 54.2 (SD 12.6) yrs and 23 age-matched controls | T1-weighted at 3T | Correlation between actual and predicted scores 0.66 (RMSE 0.79) |
| Deep 3D convolutional encoder networks with shortcut connections | Lesion segmentation | WMH | [16]  [10.1109/TMI.2016.2528821](https://doi.org/10.1109/TMI.2016.2528821) | 64 datasets from MICCAI 2008 and ISBI 2015 challenges | T1-, T2-weighted and FLAIR | Mean Dice 49.2-63.8 |
| Combination of morphological filters, discrete wavelet transform and Fuzzy C-means clustering (semi-automatic) | Lesion detection | Ischaemic lesions | [17]  10.1016/j.ejmp.2016.07.249 | 10 non-enhanced CT datasets | CT scans | Difference with ground truth <=12% |
| Convolutional neural networks trained with non-uniformly sampled patches | Lesion segmentation | WMH | [18]  [10.1109/ISBI.2016.7493532](https://doi.org/10.1109/ISBI.2016.7493532) | 466 datasets from the RUN DMC Study (DOI: 10.1186/1471-2377-11-29) : non-demented elderly aged 50-85 yrs | T1-weighted and FLAIR | Mean Dice 0.78 |
| 1)Subtraction of the z-scores of the normalised intensities, 2)Generalised likelihood ratio of the normalised intensities in the WM, 3)Logistic regression model with multiple image modalities | Lesion change detection | Ischaemic lesions | [19]  10.1007/s12021-016-9301-1 | 20 Multiple sclerosis patients | T1-, T2- weighted and FLAIR | Mean Dice coefficient 0.3-0.58 |
| Support-vector machine to calculate: SPARE-BA index (capturing age-related brain atrophy), and the SPARE-AD index (developed to capture patterns of atrophy found in patients with Alzheimer’s disease in [10.1109/TMI.2006.886812](https://doi-org.ezproxy.is.ed.ac.uk/10.1109/TMI.2006.886812)). | Study age-related and atrophy-related patterns | Ischaemic lesions and atrophy | [20]  [10.1093/brain/aww008](https://doi.org/10.1093/brain/aww008) | 2367 adults ages 20-90 years from the Study of Health in Pomerania cohort 10.1007/s00103-012-1483-6 | T1-weighted and FLAIR at 1.5T | ROC 0.88 and 0.92 on the datasets used to develop the method. Methods not tested in this sample |
| Wavelet-based fusion of CT and DWI co-registered images, segmentation using k-means and post-processing using morphological operators | Lesion detection | Acute ischaemic stroke lesion | [21]  [10.1109/ICACCI.2015.7275761](https://doi.org/10.1109/ICACCI.2015.7275761) | 18 datasets | DWI and a CT scan | Stroke correctly identified in 16/18 cases. |
| Active learning selecting sample on a random forest classifier | Lesion Segmentation | Ischaemic stroke lesion | [22]  10.1016/j.neucom.2014.01.077 | Not specified (one dataset?) | T1-,T2-weighted, FLAIR, fMRI data (ReHo,ALFF,fALFF), DWI data (AD,MD,RD,FA) | Accuracy close to 100%, sensitivity close to 90% |
| Haralick 14 textural features extracted and input to a Support Vector Machine classifier (linear, quadratic and RBF kernels evaluated) | Lesion detection, segmentation and stroke-subtype classification | Stroke lesion | [23]  [10.1109/ICCC.2015.7432922](http://dx.doi.org/10.1109/ICCC.2015.7432922) | 2 datasets | CT scans | Accuracy 89-91%, Sensitivity 85-92% Specificity 86-89% |
| 1)Unified segmentation normalization to standard space and generate tissue probability maps, 2) Non-rigid and reversible atlas-based registration to refine the probability maps, 3) Combine probability maps with the normalized MRI to construct three types of features, used to train three support vector machine (SVM) classifiers for a combined classifier to detect lesion. | Lesion segmentation | Ischaemic lesions | [24]  10.1186/s12880-015-0092-x | 60 stroke patients mean age 61.6 (SD 12.27) yrs | 3D T1-MPRAGE, T2-weighted , at 3T | Accuracy 97.6-98%, mean Dice 0.51-0.66, sensitivity (recall) 59-67%, precision 47-66.5% |
| Removal of pixels with brightness levels of cerebrospinal  Fluid. Threshold remaining pixels separating healthy and stroke tissues | Lesion segmentation | Ischaemic cortical strokes | [25]  10.1007/s10527-015-9522-x | 7 datasets | FLAIR | Mean volume difference 5.51% |
| (1) Three statistical *a-priori* tissue atlases (CSF, GM and WM) and a brain structure atlas are registered to the patient space and used to (2) guide the tissue segmentation on T1-w (3) the output segmentation is used to detect and refill WM outliers as normal-appearing WM based on the registered *a-priori* and hyper-intense FLAIR maps if available. The voxel intensities of candidate regions on T1-w are refilled with normal-appearance WM intensities (4) the tissue is re-estimated again (5) intermediate volume maps are reassigned into CSF, GM and WM using both neighbour and spatial prior information. | Segmentation | WMH | [26]  [10.1016/j.media.2016.08.014](http://dx.doi.org/10.1016/j.media.2016.08.014) | 20 scans with varying degrees of brain atrophy and white matter lesions from the MRBrainS 2013 database <http://mrbrains13.isi.uu.nl/> | T1-weighted, FLAIR at 3T | True lesions detected 33% to 41%. (Segmentation of other tissues more accurate) |
| Automatic and supervised learning: LINDA (Lesion Identification with Neighborhood Data Analysis): 12 features extracted from T1-w used in a multi-resolution voxel-neighborhood random forest algorithm (“mrvnrf” command in ANTsR). | Segmentation | Large Cortical ischaemicstroke lesions | [27]  10.1002/hbm.23110 | 60 left hemispheric chronic stroke patients (age: 57.2 ± 11.5 yrs, post-stroke interval: 2.6 ± 2 yrs), 80 age-matched controls, and 45 patients (age: 59.6 ± 9.5 years, post-stroke interval: 3.6 ± 3.1) for validation | T1-weighted acquired with a 3D inversion recovery sequence at 3T | Mean Dice 0.696 ± 0.16, Hausdorff distance 17.9 ± 9.8 mm, and mean displacement 2.54 ± 1.4 mm |
| Semi-automatic computer-aided CMB detection system that uses supervised learning: (i) candidates screening based on intensity values (ii) compact 3D hierarchical features extraction via a stacked convolutional Independent Subspace Analysis (ISA) network (iii) false positive candidates removal with a support vector machine (SVM) classifier based on the learned representation features from ISA | Feature detection | Brain micro-bleeds | [28]  10.1109/EMBC.2015.7320232 | 44 patients (25 training and 19 testing) | SWI at 3T | AUC 0.9, Sensitivity 89.4%, Precision 49.7% |
| 3D Convolutional Neural Networks | Feature detection | Brain micro-bleeds | [29]  [10.1109/TMI.2016.2528129](https://doi.org/10.1109/TMI.2016.2528129) | 320: 126 stroke patients (mean age 67.4±11.3 yrs) and 194 normal elderly (mean age 71.2±5.0 yrs) | SWI at 3T | Sensitivity 91.4-92.3%, precision 27.2-42.7%, false positives ave. 2.9 |
| Deep learning based 3D feature representation: 1) CMB candidates localization by statistical thresholding, 2) the deep convolutional neural network (CNN) for hierarchical 3D feature representation, 3) the SVM classifier is trained on the features to distinguish true BMB-s and BMB mimics. | Feature detection | Brain micro-bleeds | [30]  [10.1109/ISBI.2015.7163984](http://dx.doi.org/10.1109/ISBI.2015.7163984) | 20 elderly subjects (mean age 78.6) with transient ischemic attack | SWI at 3T | Sensitivity 89.2%, precision 56.2%, false positives ave. 6.4 |
| Regions with microbleeds are identified by a multi-scale Laplacian of Gaussian. A cascade of random forest classifiers that uses Radon- and Hessian-based features handles class imbalance problem | Feature detection | Brain micro-bleeds | [31]  [10.1016/j.compmedimag.2015.10.001](http://dx.doi.org/10.1016/j.compmedimag.2015.10.001) | 66 subjects | SWI at 3T | Sensitivity 87-97% |
| Random Forest in cascade learning using 2 features from patches: center location of the patch, and intensity information of the patch from 3D Haar-like operators. | Atlas-based Segmentation | ROIs from atlases | [32]  [10.1016/j.patcog.2016.09.019](http://dx.doi.org/10.1016/j.patcog.2016.09.019) | 16 subjects | T1-weighted | Mean Dice 0.7-0.9 and SSIM 0.93 |
| Sliding window approach and a multi-class random forest classifier applied to high-dimensional feature vectors (SEGMA) | Atlas-based Segmentation | ROIs, main tissues | [33]  [10.3389/fninf.2017.00002](https://doi.org/10.3389/fninf.2017.00002) | 179 individuals, from three age groups: newborns (38–42 weeks gestational age), children and adolescents (4–17 years) and adults (35–71 years). | T1-weighted | Mean Dice 0.8-0.9 |
| Pairwise Markov Random Field, where registration and segmentation nodes are coupled towards simultaneously recovering all atlas deformations and labelling the query image | Atlas-based Segmentation | ROIs | [34]  10.1007/s11263-016-0925-2 | 18 datasets from the Internet Brain Segmentation Repository (IBSR). [10.1109/TMI.2011.2163944](https://doi.org/10.1109/TMI.2011.2163944) | T1-weighted | Mean Dice 0.77, and  Symmetric Mean Surface Distance (SMSD) 1.01 mm |
| Acceleration of 3D Otsu algorithm using dimension decomposition rule. The whole segmentation algorithm is designed as an iteration procedure. In each iteration, the image is segmented by 3D Otsu, and then it is filtered by a fast local Laplacian filtering to get a smoothed image which will be input into the next iteration. Finally, the segmentation results are pooled to get a final segmentation using majority voting rules. | Threshold-based segmentation | Not specified | [35]  [10.1016/j.dsp.2016.08.003](http://dx.doi.org/10.1016/j.dsp.2016.08.003) | Examples from <http://www.med.harvard.edu/aanlib/home.html> (n not specified) | CT, T2-weighted | Visual approval by experts and reduction time to half the conventional Otsu thresholding |
| (1) segmentation of the brain parenchyma, (2) automated detection step for initial WMH regions, (3) subsequent semi-automated re-segmentation step for the WMH regions, and (4) determination of the WMH volume ratio. | Segmentation | WMH | [36]  10.1117/12.845229 | 10 patients with a diagnosis of VaD | FLAIR | Mean Dice 78.2±11.0% |
| Deep learning model (CNN) of tissue fate based on randomly sampled local patches from the hypoperfusion (Tmax) feature observed in MRI immediately after symptom onset | Evolution prediction | Ischaemic stroke lesion | [37]  [10.1109/BIBM.2015.7359869](https://doi.org/10.1109/BIBM.2015.7359869) | 25 stroke patients, but perfusion successful in 19/25 | FLAIR and perfusion (Tmax) imaging | Mean accuracy 85.3+/-9.1 % |
| Infarct semi-automatically segmented at baseline and follow-up. Perfusion and diffusion processed using toolbox AnToNIa 10.3414/ME14-01-0007 | Evolution prediction | Ischaemic stroke lesion | [38]  10.1117/12.2082686 | 23 MRI datasets of acute stroke patients with known tissue outcome | FLAIR, DWI, perfusion imaging | Mean Dice 0.45 |
| From four classifiers evaluated using texture attributes extracted from manually selected ROIs best results were obtained from the support vector machine classifier with a radial based function kernel. The most discriminating texture attributes were obtained from the gray-level histogram and from the co-occurrence matrix. | Aetiology-based classification | WMH | [39]  [10.1117/1.JMI.2.1.014002](https://dx.doi.org/10.1117%2F1.JMI.2.1.014002) | 54 patients diagnosed with MS or stroke and 19 normal volunteers | T2-weighted at 1.5T | Mean accuracy 87.9% distinguishing lesions with different aetiologies and accuracy of 99.29% distinguishing normal white matter from WMH |
| Extra Tree forest framework for voxel-wise classification mainly using intensity derived image features. | Segmentation | Sub-acute ischaemic stroke lesion | [40]  10.1016/j.jneumeth.2014.11.011 | 37 clinical cases | FLAIR, T1-, T2-weighted and DWI at 3T | Mean Dice 0.63-0.7 |
| Automatic framework based on multiple instance learning (MIL). Intensity patches are extracted from regions with high probability of containing lesions. These are then used as instances in MIL for the identification of SVD. | Classification, distinguishing between absent/mild SVD and moderate/ severe SVD | Hyper-attenuated lesions | [41]  10.1007/978-3-319-24553-9_64 | 590 stroke patients mean age 70.7 (SD 10.8) years | CT scans | 75% accuracy, 80% sensitivity and 70% specificity |
| Improvement of the CAD scheme proposed in [43] by using kernel eigenspace template matching. After the ROIs around the candidate regions detected in [43] are selected, a kernel eigenspace is made by using kernel principal component analysis of the training data set. A test ROI is projected onto the same kernel eigenspace as the training data set. The cross-correlation coefficients between the test ROI and all the training ROIs are calculated on the kernel eigenspace. By comparing the two maxima of coefficients with a lacunar ROI and an FP ROI, the test ROI is classified. | Feature detection | Lacunar infarcts | [42]  10.6009/jjrt.2015_JSRT_71.2.85 | 30 patients: 15 with and 15 without lacunar infarcts | T2-weighted | 31. 9% of false positives were eliminated |
| Spectral clustering is used to partition the space into strongly connected clusters representing subsets. With fuzzy training set sampling, overlapping local sets classifiers are subsequently trained. | Lesion Segmentation | Ischaemic cortical stroke lesions | [44]  [10.1109/ISBI.2015.7163994](http://dx.doi.org/10.1109/ISBI.2015.7163994) | 37 stroke patients | FLAIR | Mean Dice 0.56 |
| Multiclass Support Vector Machine (SVM)-Based Lesion Mapping. Previously segmented infarct regions are used to calculate the percentage of lesioned voxels in the predefined MNI, Harvard-Oxford cortical and subcortical atlas regions, and to generate four problem-specific VOIs, identified from the database using voxel-based lesion symptom mapping. An overall of 12 SVM classification models for predicting the corresponding mRS score are generated using the lesion overlap values from the different brain region definitions, stroke laterality information, and the optional parameters infarct volume, admission NIHSS, and patient age. | Predict functional outcome in ischaemic stroke | Lesions | [45]  [10.1371/journal.pone.0129569](http://dx.doi.org/10.1371/journal.pone.0129569) | 68 stroke patients median age 71.5yrs, median follow-up imaging time 34.5 days | FLAIR | Prediction accuracy 56% and binary prediction accuracy 85% |
| Automated voxel-based classification that uses sequential random forest classifiers trained with a number of randomized 3D Haar features. | Segmentation | Peri-vascular spaces | [46]  [10.1016/j.neuroimage.2016.03.076](http://dx.doi.org/10.1016/j.neuroimage.2016.03.076) | 17 healthy volunteers aged from 25 to 37 | T1-weighted MP2RAGE, T2-weighted at 7T | Mean Dice 0.55-0.65 |
| Supervised voxel-based random forest classification CAD system, optimized and trained to exclusively detect small WMLs. Pre-processing steps included standardization of subject intensities. Features calculated included multimodal intensities, tissue probabilities, several features for accurate location description, a number of second order derivative features as well as multi-scale annular filter for blobness detection. | Small lesion detection | WMH | [47]  10.1117/12.2081597 | 503 SVD patients with MCI evidence. 32 cases used for testing | 3D T1-weighted, FLAIR, T2* GRE | ROC curves shown |
| Four boosting methods are compared with two other classifiers: SVM and random forest in (i) Segmentation of potential areas with distorted brain tissue (selection of regions of interest), (ii) acute stroke tissue recognition by extracting and then classifying a set of well-differentiating features. Descriptors determined in several image transformation domains: 2D Fourier domain, polar 2D Fourier domain, and multiscale domains (i.e., wavelet, complex wavelet, and contourlet domain). | Pattern recognition | Stroke lesions | [48]  10.1007/978-3-319-26227-7_8 | 8 normal and 32 stroke patients | CT scans | Accuracy close to 75% |
| Automatic naïve Bayes classification scheme. Probabilistic tissue segmentation and image algebra create feature maps encoding information about missing and abnormal tissue. | Pattern recognition | Ischaemic stroke lesions | [49]  10.1016/j.jneumeth.2015.09.019 | 30 cases with left hemisphere stroke lesions | T1-weighted | Mena Dice 0.66 |

**References to Table S1**

1 Gonzalez-Castro V, Valdés Hernández MC, Armitage PA, Wardlaw JM: Automatic Rating of Perivascular Spaces in Brain MRI Using Bag of Visual Words. Lecture Notes in Computer Science 2016;9730:642-649.

2 Viksne L, Valdés Hernández MC, Hoban K, Heye AK, Gonzalez-Castro V, Wardlaw JM: Textural Characterisation on Regions of Interest: A Useful Tool for the Study of Small Vessel Disease; in Dr.Tryphon Lambrou and Dr.Xujiong Ye, (ed): University of Lincoln, UK, www.miua.org.uk, sponsored by British Machine Vision Association, 2015, pp 66-71. https://www.semanticscholar.org/paper/Textural-Characterisation-on-Regions-of-Interest-A-Viksne-Hern%C3%A1ndez/534fadacdab167d128ea35e61fad81abe075ff18

3 Gonzalez-Castro V, Valdés Hernández MC, Armitage PA, Wardlaw JM: Texture-based classification for the automatic rating of the perivascular spaces in brain MRI. Procedia Computer Science 2016;90:9-14.

4 Ballerini L, Lovreglio R, Valdés Hernández MC, Gonzalez-Castro V, Muñoz Maniega S, Pellegrini E, Bastin M, Deary I, Wardlaw JM: Application of the ordered logit model to optimising Frangi filter parameters for segmentation of perivascular spaces. Procedia Computer Sciences 2016;90:61-67.

5 Gao XW, Hui R: A deep learning based approach to classification of CT brain images . SAI Computing Conference (SAI), 2016 2016;28-31.

6 Wang Y, Katsaggelos AK, Wang X, Parrish TB: A deep symmetry convnet for stroke lesion segmentation. 2016 IEEE International Conference on Image Processing (ICIP), Phoenix, AZ, 2016 2016;111-115.

7 B. H. Menze et al., "A Generative Probabilistic Model and Discriminative Extensions for Brain Lesion Segmentation— With Application to Tumor and Stroke," in IEEE Transactions on Medical Imaging, vol. 35, no. 4, pp. 933-946, April 2016. doi: 10.1109/TMI.2015.2502596

8 K. Zeng, G. Erus, A. Sotiras, R. T. Shinohara and C. Davatzikos, "Abnormality Detection via Iterative Deformable Registration and Basis-Pursuit Decomposition," in IEEE Transactions on Medical Imaging, vol. 35, no. 8, pp. 1937-1951, Aug. 2016. doi: 10.1109/TMI.2016.2538998

9 Pedro Costa Klein, Ricardo Bernardi Soder, Jefferson Becker, Alexandre Rosa Franco, and Márcio Sarroglia Pinho. 2016. An automatic method for multiple sclerosis lesion detection in fluid attenuated inversion recovery magnetic resonance images. In Proceedings of the 31st Annual ACM Symposium on Applied Computing (SAC '16). ACM, New York, NY, USA, 305-308. DOI: 10.1145/2851613.2851938

10 Jayaram, P.V., Menaka, R.An experimental study of Stockwell transform-based feature extraction method for ischemic stroke detection (2016) International Journal of Biomedical Engineering and Technology, 21 (1), pp. 40-48. https://www.scopus.com/inward/record.uri?eid=2-s2.0-84974737255&partnerID=40&md5=1814054d62bd905e41fb8d611afc282e

11 T.L.A. van den Heuvel, A.W. van der Eerden, R. Manniesing, M. Ghafoorian, T. Tan, T.M.J.C. Andriessen, T. Vande Vyvere, L. van den Hauwe, B.M. ter Haar Romeny, B.M. Goraj, B. Platel, Automated detection of cerebral microbleeds in patients with traumatic brain injury, NeuroImage: Clinical, Volume 12, February 2016, Pages 241-251, ISSN 2213-1582, http://dx.doi.org/10.1016/j.nicl.2016.07.002, (http://www.sciencedirect.com/science/article/pii/S221315821630122X)

12 Alex M. Pagnozzi, Nicholas Dowson, James Doecke, Simona Fiori, Andrew P. Bradley, Roslyn N. Boyd, Stephen Rose, Automated, quantitative measures of grey and white matter lesion burden correlates with motor and cognitive function in children with unilateral cerebral palsy, NeuroImage: Clinical, Volume 11, 2016, Pages 751-759, ISSN 2213-1582, http://dx.doi.org/10.1016/j.nicl.2016.05.018, (http://www.sciencedirect.com/science/article/pii/S2213158216300948)

13 Ludovica Griffanti, Giovanna Zamboni, Aamira Khan, Linxin Li, Guendalina Bonifacio, Vaanathi Sundaresan, Ursula G. Schulz, Wilhelm Kuker, Marco Battaglini, Peter M. Rothwell, Mark Jenkinson, BIANCA (Brain Intensity AbNormality Classification Algorithm): A new tool for automated segmentation of white matter hyperintensities, NeuroImage, Volume 141, 1 November 2016, Pages 191-205, ISSN 1053-8119, http://dx.doi.org/10.1016/j.neuroimage.2016.07.018, (http://www.sciencedirect.com/science/article/pii/S1053811916303251)

14 Teh, V., Sim, K. S. and Wong, E. K. (2016), Brain early infarct detection using gamma correction extreme-level eliminating with weighting distribution. Scanning, 38: 842–856. doi:10.1002/sca.21334

15 Jane M. Rondina, Maurizio Filippone, Mark Girolami, Nick S. Ward, Decoding post-stroke motor function from structural brain imaging, NeuroImage: Clinical, Volume 12, February 2016, Pages 372-380, ISSN 2213-1582, http://dx.doi.org/10.1016/j.nicl.2016.07.014, (http://www.sciencedirect.com/science/article/pii/S2213158216301346)

16 T. Brosch, L. Y. W. Tang, Y. Yoo, D. K. B. Li, A. Traboulsee and R. Tam, "Deep 3D Convolutional Encoder Networks With Shortcuts for Multiscale Feature Integration Applied to Multiple Sclerosis Lesion Segmentation," in IEEE Transactions on Medical Imaging, vol. 35, no. 5, pp. 1229-1239, May 2016.doi: 10.1109/TMI.2016.2528821

17 Allan F.F. Alves, Ana J.M. Sampaio, Nadine H.P.B. Maues, Marcela de Oliveira, Ana L.M. Pavan], Carlos C.M. de Freitas, Nitamar Abdala, D.R. Pina, Ischemic stroke detection in non-enhanced computed tomography examinations, Physica Medica, Volume 32, Supplement 3, September 2016, Page 335, ISSN 1120-1797, http://dx.doi.org/10.1016/j.ejmp.2016.07.249, (http://www.sciencedirect.com/science/article/pii/S1120179716303829)

18 M. Ghafoorian et al., "Non-uniform patch sampling with deep convolutional neural networks for white matter hyperintensity segmentation," 2016 IEEE 13th International Symposium on Biomedical Imaging (ISBI), Prague, 2016, pp. 1414-1417, doi: 10.1109/ISBI.2016.7493532

19 Lesjak, Ž., Pernuš, F., Likar, B. et al. Neuroinform (2016) 14: 403. doi:10.1007/s12021-016-9301-1

20 Mohamad Habes, Guray Erus, Jon B. Toledo, Tianhao Zhang, Nick Bryan, Lenore J. Launer, Yves Rosseel, Deborah Janowitz, Jimit Doshi, Sandra Van der Auwera, Bettina von Sarnowski, Katrin Hegenscheid, Norbert Hosten, Georg Homuth, Henry Völzke, Ulf Schminke, Wolfgang Hoffmann, Hans J. Grabe, Christos Davatzikos; White matter hyperintensities and imaging patterns of brain ageing in the general population. Brain 2016; 139 (4): 1164-1179. doi: 10.1093/brain/aww008

21 P. R. Mirajkar, K. A. Bhagwat, A. Singh and Ashalatha M E, "Acute ischemic stroke detection using wavelet based fusion of CT and MRI images," 2015 International Conference on Advances in Computing, Communications and Informatics (ICACCI), Kochi, 2015, pp. 1123-1130.doi: 10.1109/ICACCI.2015.7275761

22 Darya Chyzhyk, Rosalía Dacosta-Aguayo, Maria Mataró, Manuel Graña, An active learning approach for stroke lesion segmentation on multimodal MRI data, Neurocomputing, Volume 150, Part A, 20 February 2015, Pages 26-36, ISSN 0925-2312, http://dx.doi.org/10.1016/j.neucom.2014.01.077, (http://www.sciencedirect.com/science/article/pii/S0925231214012466)

23 Jeena R S and S. Kumar, "Analysis of Stroke using texture features," 2015 International Conference on Control Communication & Computing India (ICCC), Trivandrum, 2015, pp. 366-370, doi: 10.1109/ICCC.2015.7432922

24 Guo D, Fridriksson J, Fillmore P et al. Automated lesion detection on MRI scans using combined unsupervised and supervised methods BMC Medical Imaging 2015 15:50, DOI: 10.1186/s12880-015-0092-x

25 Dmitriev G A, Kirsanova A V, Al-Baheli W A. Automated Recognition of Cerebral Stroke Boundaries Based on Threshold Processing of Magnetic Resonance Images. Biomed Eng 2015, 49(3):168-170. doi:10.1007/s10527-015-9522-x

26 Sergi Valverde, Arnau Oliver, Eloy Roura, Sandra González-Villà, Deborah Pareto, Joan C. Vilanova, Lluís Ramió-Torrentà, Àlex Rovira, Xavier Lladó, Automated tissue segmentation of MR brain images in the presence of white matter lesions, Medical Image Analysis, Volume 35, January 2017, Pages 446-457, ISSN 1361-8415, http://dx.doi.org/10.1016/j.media.2016.08.014, (http://www.sciencedirect.com/science/article/pii/S1361841516301621)

27 Pustina, D., Coslett, H. B., Turkeltaub, P. E., Tustison, N., Schwartz, M. F. and Avants, B. (2016), Automated segmentation of chronic stroke lesions using LINDA: Lesion identification with neighborhood data analysis. Hum. Brain Mapp., 37: 1405–1421. doi:10.1002/hbm.23110

28 Q. Dou et al., "Automatic cerebral microbleeds detection from MR images via Independent Subspace Analysis based hierarchical features," 2015 37th Annual International Conference of the IEEE Engineering in Medicine and Biology Society (EMBC), Milan, 2015, pp. 7933-7936, doi: 10.1109/EMBC.2015.7320232

29 Q. Dou et al., "Automatic Detection of Cerebral Microbleeds From MR Images via 3D Convolutional Neural Networks," in IEEE Transactions on Medical Imaging, vol. 35, no. 5, pp. 1182-1195, May 2016, doi: 10.1109/TMI.2016.2528129

30 H. Chen, L. Yu, Q. Dou, L. Shi, V. C. T. Mok and P. A. Heng, "Automatic detection of cerebral microbleeds via deep learning based 3D feature representation," 2015 IEEE 12th International Symposium on Biomedical Imaging (ISBI), New York, NY, 2015, pp. 764-767, doi: 10.1109/ISBI.2015.7163984

31 Amir Fazlollahi, Fabrice Meriaudeau, Luca Giancardo, Victor L. Villemagne, Christopher C. Rowe, Paul Yates, Olivier Salvado, Pierrick Bourgeat, Computer-aided detection of cerebral microbleeds in susceptibility-weighted imaging, Computerized Medical Imaging and Graphics, Volume 46, Part 3, December 2015, Pages 269-276, ISSN 0895-6111, http://dx.doi.org/10.1016/j.compmedimag.2015.10.001, (http://www.sciencedirect.com/science/article/pii/S0895611115001421)

32 Jinpeng Zhang, Lichi Zhang, Lei Xiang, Yeqin Shao, Guorong Wu, Xiaodong Zhou, Dinggang Shen, Qian Wang, Brain atlas fusion from high-thickness diagnostic magnetic resonance images by learning-based super-resolution, Pattern Recognition, Volume 63, March 2017, Pages 531-541, ISSN 0031-3203, http://dx.doi.org/10.1016/j.patcog.2016.09.019, (http://www.sciencedirect.com/science/article/pii/S0031320316302825)

33 Serag Ahmed, Wilkinson Alastair G., Telford Emma J., Pataky Rozalia, Sparrow Sarah A., Anblagan Devasuda, Macnaught Gillian, Semple Scott I., Boardman James P. SEGMA: An Automatic SEGMentation Approach for Human Brain MRI Using Sliding Window and Random Forests Frontiers in Neuroinformatics (2017), 11, DOI=10.3389/fninf.2017.00002

34 Alchatzidis, S., Sotiras, A., Zacharaki, E.I. et al. Int J Comput Vis (2017) 121: 169. doi:10.1007/s11263-016-0925-2

35 Yuncong Feng, Haiying Zhao, Xiongfei Li, Xiaoli Zhang, Hongpeng Li, A multi-scale 3D Otsu thresholding algorithm for medical image segmentation, Digital Signal Processing, Volume 60, January 2017, Pages 186-199, ISSN 1051-2004, http://dx.doi.org/10.1016/j.dsp.2016.08.003, (http://www.sciencedirect.com/science/article/pii/S1051200416301191)

36 Hidetaka Arimura ; Yasuo Kawata ; Yasuo Yamashita ; Taiki Magome ; Masafumi Ohki ; Fukai Toyofuku ; Yoshiharu Higashida and Kazuhiro Tsuchiya"Computerized evaluation method of white matter hyperintensities related to subcortical vascular dementia in brain MR images", Proc. SPIE 7624, Medical Imaging 2010: Computer-Aided Diagnosis, 762424 (March 09, 2010); doi:10.1117/12.845229; http://dx.doi.org/10.1117/12.845229

37 N. Stier, N. Vincent, D. Liebeskind and F. Scalzo, "Deep learning of tissue fate features in acute ischemic stroke," 2015 IEEE International Conference on Bioinformatics and Biomedicine (BIBM), Washington, DC, 2015, pp. 1316-1321, doi: 10.1109/BIBM.2015.7359869

38 Nils Daniel Forkert and Jens Fiehler "Effect of sample size on multi-parametric prediction of tissue outcome in acute ischemic stroke using a random forest classifier ", Proc. SPIE 9417, Medical Imaging 2015: Biomedical Applications in Molecular, Structural, and Functional Imaging, 94172H (March 17, 2015); doi:10.1117/12.2082686

39 Leite M, Rittner L, Appenzeller S, Ruocco HH, Lotufo R. Etiology-based classification of brain white matter hyperintensity on magnetic resonance imaging. Journal of Medical Imaging. 2015;2(1):014002. doi:10.1117/1.JMI.2.1.014002

40 Oskar Maier, Matthias Wilms, Janina von der Gablentz, Ulrike M. Krämer, Thomas F. Münte, Heinz Handels, Extra Tree forests for sub-acute ischemic stroke lesion segmentation in MR sequences, Journal of Neuroscience Methods, Volume 240, 30 January 2015, Pages 89-100, ISSN 0165-0270, http://dx.doi.org/10.1016/j.jneumeth.2014.11.011, (http://www.sciencedirect.com/science/article/pii/S0165027014004038)

41 Chen L. et al. (2015) Identification of Cerebral Small Vessel Disease Using Multiple Instance Learning. In: Navab N., Hornegger J., Wells W., Frangi A. (eds) Medical Image Computing and Computer-Assisted Intervention -- MICCAI 2015. Lecture Notes in Computer Science, vol 9349. Springer, Cham

42 Murakawa S, Tanigawa A, Uchiyama Y, Muramatsu C, Hara T, Fujita H. Kernel eigenspace template matching for detection of lacunar infarcts on MR images. Nihon Hoshasen Gijutsu Gakkai Zasshi. 2015 Feb;71(2):85-91. doi: 10.6009/jjrt.2015_JSRT_71.2.85

43 Uchiyama, Y., Asano, T., Kato, H. et al. J Digit Imaging (2012) 25: 497. doi:10.1007/s10278-011-9444-4

44 O. Maier and H. Handels, "Local problem forests: Classifier training for locally limited sub-problems using spectral clustering," 2015 IEEE 12th International Symposium on Biomedical Imaging (ISBI), New York, NY, 2015, pp. 806-809, doi: 10.1109/ISBI.2015.7163994

45 Forkert ND, Verleger T, Cheng B, Thomalla G, Hilgetag CC, et al. (2015) Multiclass Support Vector Machine-Based Lesion Mapping Predicts Functional Outcome in Ischemic Stroke Patients. PLOS ONE 10(6): e0129569. doi: 10.1371/journal.pone.0129569

46 Sang Hyun Park, Xiaopeng Zong, Yaozong Gao, Weili Lin, Dinggang Shen, Segmentation of perivascular spaces in 7 T MR image using auto-context model with orientation-normalized features, NeuroImage, Volume 134, 1 July 2016, Pages 223-235, ISSN 1053-8119, http://dx.doi.org/10.1016/j.neuroimage.2016.03.076, (http://www.sciencedirect.com/science/article/pii/S1053811916300209)

47 Mohsen Ghafoorian ; Nico Karssemeijer ; Inge van Uden ; Frank E. de Leeuw ; Tom Heskes ; Elena Marchiori ; Bram Platel; Small white matter lesion detection in cerebral small vessel disease. Proc. SPIE 9414, Medical Imaging 2015: Computer-Aided Diagnosis, 941411 (March 20, 2015); doi:10.1117/12.2081597

48 Ostrek G., Nowakowski A., Jasionowska M., Przelaskowski A., Szopiński K. (2016) Stroke Tissue Pattern Recognition Based on CT Texture Analysis. In: Burduk R., Jackowski K., Kurzyński M., Woźniak M., Żołnierek A. (eds) Proceedings of the 9th International Conference on Computer Recognition Systems CORES 2015. Advances in Intelligent Systems and Computing, vol 403. Springer, Cham

49 Joseph C. Griffis, Jane B. Allendorfer, Jerzy P. Szaflarski, Voxel-based Gaussian naïve Bayes classification of ischemic stroke lesions in individual T1-weighted MRI scans, Journal of Neuroscience Methods, Volume 257, 15 January 2016, Pages 97-108, ISSN 0165-0270, http://dx.doi.org/10.1016/j.jneumeth.2015.09.019, (http://www.sciencedirect.com/science/article/pii/S0165027015003520)
