# Supplementary material for: Advanced Neuroimaging of Cerebral Small Vessel Disease
Source: Curr Treat Options Cardiovasc Med. 2017 Jun 15;19(7):56. doi: 10.1007/s11936-017-0555-1 (PMC5486578; doi:10.1007/s11936-017-0555-1)
Supplement: Supplementary file 2 — (DOC 322 kb). [file 11936_2017_555_MOESM2_ESM.doc]

**Table S2:** Original papers published since 1^st^ January 2015 on White Matter Integrity and SVD

| Study | N | Age | SVD Group | Control Group | Imaging Method | Variables Controlled For | Main Findings |
| --- | --- | --- | --- | --- | --- | --- | --- |
| Baykara et al. 2016(1) | 170 CADASIl  549 sporadic SVD  37 AD  89 MCI  241 Healthy controls | From two cohorts:  49.1(9.5) and 53.4(10.7)  From two cohorts:  65.3(8.9) and 74.9(8.3)  74 (8.2)  From two cohorts:  76.5 (4.4) and 74.7(8.1)  From three cohorts:  71.5(6.3) 72.9(5.7) and 66.9(11.4) | 2x CADASIL cohorts  Sporadic SVD: Neuroimaging defined cohort and memory clinic vascular cognitive impairment patient cohort | AD:  Alzheimer’s disease subjects from ADNI  MCI:  Subjects from ADNI and subjects with presumed AD type MCI from VASCAMY study  Healthy controls:  Healthy controls from ADNI, VASCAMy and Austrian stroke prevention study | 1.5T and 3T DTI | Age, sex, normalised WMH volume, lacune volume, brain parenchymal fraction and microbleed count. | PSMD was increased in SVD samples and increased with higher white matter load. PSMD explained the largest amount of variance in processing speed in SVD samples compared to other imaging markers of SVD. In non-SVD samples including patients with Alzheimer’s disease, PSMD was not associated with processing speed. In a longitudinal analysis of 58 CADASIL patients PSMD and WMH volume were seen to change over 18 months follow up whereas other markers and processing speed did not. PSMD had the smallest sample size for a clinical trial based on the changes seen in this sample. |
| Chen et al. 2015(2) | 27 subjects  30 controls | 65.8±5.9  63.5±6.1 | Silent lacunar infarcts on MRI in cognitively healthy cohort | Participants from the same cohort without silent lacunar infarcts | 3T DTI | Age, sex and education | FA was decreased and MD increased in the corpus callosum and superior and anterior corona radiata compared to controls. Memory, executive function, language scores and MMSE were also reduced. Silent lacunar infarct patients with higher FA in the left external capsule had higher cognitive test scores in memory and language domains. Presence of other SVD features was not commented on. |
| Evans et al. 2016(3) | 4259 | 63.3 (11.0) | Stroke free community dwelling cohort | None | 1.5T DTI | Age, sex, cardiovascular risk factors, WMH volume and lacunar infarcts | Each SD decrease in FA associated with a hazard ratio of stroke of 0.75 (0.57-0.98) and each SD increase in MD associated with a hazard ratio of stroke 1.50 (1.08-2.09). |
| Hollocks et al. 2015(4) | 118 subjects  398 controls | 69.9 (9.8)  61.9 (13.5) | Symptomatic lacunar stroke plus Fazekas grade 2 or higher WMH | Stroke free healthy controls (some vascular risk factors present) | 1.5T DTI | Age, IQ, global cognitive function | Decreased FA was associated with apathy but not depression. The strongest associations were seen in limbic association tracts. Analysis did not take into account WMH volume. |
| Kim et al. 2015(5) | 232 | 73.7 (7.2)  74.1 (6.6)  69.7 (9.5)  70.1(7.9) | Mixed cohort of subcortical and Alzheimer’s type dementia and MCI | None | 3T DTI  PiB-PET imaging | Age, sex, education, PiB retention ratio, WMH volume and lacune number | WMH volume and lacunes were associated with decreased nodal efficiency across frontal, lateral temporal, lateral parietal and occipital regions (nodal efficiency is essentially a measure of the path length between a node and all the other nodes in a network; shorter path lengths are more efficient, and a decrease in nodal efficiency means communications are being routed by via a longer path). Using path analysis frontal nodal efficiency appeared to mediate the effects of SVD on frontal atrophy and frontal executive dysfunction. Temporal and parietal nodal efficiency appeared to mediate the effects of SVD on temporal and parietal atrophy and memory dysfunction |
| Kim et al. 2016(6) | 129 | 73.8(6.8) | Subcortical vascular dementia and MCI | None | 3T DTI  PiB-PET | Age, sex, intracranial volume, clinical diagnosis, periventricular WMH and brainstem microbleeds (the imaging markers associated with gait score on univariate analysis). | Poorer gait score associated with periventricular WMH, decreased FA in frontal and parietal WM and the corpus callosum, and cortical thinning in frontal and lateral temporo-parietal-occipital areas. Amyloid assessed by Pitsburgh B imaging not related to gait score. Using path analysis the relationship of PVWMH to gait score appeared mediated via reductions in FA and cortical thickness |
| Kim et al. 2015(7) | 32 Vascular dementia  40 Alzheimer’s disease  56 Healthy Controls | 71.3±7.3  67.0±9.1  62.5±7.5 | Subcortical vascular disease patients with severe WMH amyloid negative PET imaging | Alzheimer’s disease patient’s without WMH and amyloid positive PET imaging  Neurologically and cognitively normal healthy controls | 3T DTI  PiB-PET | Age, sex and level of education | Vascular dementia patients had decreased FA and increased MD in all WM regions examined compared to AD patients without WMH. (Note the difference in WMH between groups). AD patients had decreased FA and increased MD in some WM regions compared to controls. |
| Li et al. 2016(8) | 19 subjects  15 controls | 64.7±12.4  62.1±10.1 | Subcortical stroke with no other WM pathology | None | 3T fMRI plus DTI in 13 SVD subjects | None for connectivity analyses | functional connectivity of the M1 region to motor cortex in the contralateral hemisphere was reduced post-stroke as was structural connectivity in the corpus callosum measured using fibre tracking compared to controls. After treatment with aspirin and citicoline for one month functional and structural connectivity was improved. No randomisation, blinding or untreated/placebo arm, structural connectivity only assessed in some patients and no control for demographic variable, risk factors or imaging features in the connectivity analyses. |
| Liu et al. 2015(9) | 20 subjects  16 controls | 56.8±8.4  58.8±7.3 | Lacunar stroke but excluded if any other SVD imaging features | Healthy controls | 3T fMRI and DTI | Age and sex | At 29.8 months post stroke FA decreased in stroke affected cortico-spinal tract and M1-M1 connection and increased M1-M1 functional connectivity compared to controls. |
| Maillard et la. 2016(10) | 1903 | 46±8.7 | Third generation population based cohort without prevalent stroke but unclear if this was clinical or radiological diagnosis | None | 1.5T DTI | Age, sex, use of antihypertensive medication, total cholesterol, smoking status, diabetes, total cranial volume, time between clinical and MRI assessment | Increased carotid-femoral PWV was associated with decreased FA in the corpus callosum and corona radiata and decreased GM density in the thalamus. No correction for WMH volume. |
| McEvoy et al. 2015 (11) | 173 hypertensive  101 normotensive | 61.8±2.6 | Hypertensive military veteran twins | Normotensive military veteran twins | 3T DTI | Age, site, non-independence of twin data, education level, LDL cholesterol, C-reactive protein, diabetes, statin use, alcohol use | Hypertensive subjects had decreased FA and increased MD in all WM fibre tracts. Effects were stronger in those positive for APOEe4 allele in two white matter tracts connecting frontal WM areas. Shorter duration of hypertension or better control did not lessen the effects. |
| Mutlu et al. 2016(12) | 2436 | 56.5 (6.2) | Prospective cohort of subjects aged ≥45 | None | 1.5T DTI | Age, sex, arterial or venous calibre (the calibre that was not the dependent variable), Wm volume, intracranial volume, WMH volume, lacunar infarcts, CMB, systolic blood pressure, diastolic blood pressure, antihypertensive medication, BMI, total cholesterol, HDL cholesterol, diabetes, C-reactive protein, atherosclerotic plaque and smoking | Narrower retinal arterioles and wider venules associated with decreased FA and increased MD |
| Nadkarni et al. 2016(13) | 179 | 83.1 (2.7) | Independent community dwelling adults aged 70-79 at enrolment. | None | 3T DTI | Age, sex, race, education, self reported exercise intensity, diabetes, hypertension, modified MMSE score, Parkinson’s disease rating scale (UPDRS III score), smoking status, prior stroke or TIA, grey matter volume, WMH volume and NAWM-FA(not for analyses where FA was the dependent variable) | Higher interleukin-6 levels over preceding 10 year period associated with slower gait speed and WMH volume but not NAWM FA. Association with gait speed no longer apparent after controlling for WMH volume. |
| Pasi et al. 2015(14) | 76 | 75.1±6.8 | MCI and moderate to severe WMH on Fazekas scale | None | 1.5T DTI | Age, education level, gender, lacunar infarcts, WMH, global cortical atrophy, medial temporal atrophy | Lower MoCA score but not MMSE associated with decreased FA and increased MD. Visuoexecutive and attention MoCA subsets associated with MD |
| Reijmer et al. 2015(15) | 38 subjects  29 controls |  | Non-demented subjects with Probable or definite CAA. Included 17 patients with intracerebral haemorrhage | Cognitively normal subjects without CAA | 1.5T DTI  29 subjects also had PiB PET imaging | Age, sex, education level, WMH volume, median FA, CMB, brain volume | Global network efficiency reduced in patients versus controls. Within the patient group lower global efficiency was associated with hgher cortical amyloid load, WMH volume, atrophy, processing speed, executive function and gat velocity but not associated with memory. Association of cognition with global network efficiency persisted after controlling for some demographics and SVD imaging features |
| Sam et al. 2016(16) | 45 | 74 (SD 9.4) | Patients >50 yrs presenting to neurology clinic with a range of symptoms with MRI showing WMH ≥2 on Fazekas scale | None | 3T BOLD and DTI MRI | None | CVR and FA decreased, whilst MD and T2 values increased in NAWM that progressed to WMH compared to WM in contralateral hemisphere that did not progress to WMH. |
| Sam 2016(17) | 75 | 74( SD 9.7) | Patients >50 yrs presenting to neurology clinic with a range of symptoms with MRI showing WMH ≥2 on Fazekas scale | None | 3T BOLD and DTI MRI | None | Negative CVR (suggestive of a vascular steal phenomenon) associated with decreased FA, CBF, cerebral blood volume and increased MD and time to maximum compared to regions with positive CVR |
| Santiago et al. 2015(18) | 49 | 66.3(6.8) | Cardiac rehabilitation participants with recent myocardial infarction, coronary intervention or bypass graft. Mean WMH volume 2.5ml | None | 3T DTI | Age, sex, education | Decreased FA associated with impaired processing speed and executive function in 11 out of 20 WM tracts. No association with memory in any tracts.  Increased MD associated with impaired processing speed and executive function in 9 out of 20 WM tracts. Association with memory in 3 tracts. |
| Schaapsmeerders et al. 2016(19) | 117 subjects  84 controls | 49.8±9.4  48.9±11.9 | Young ischaemic stroke patients (mean age at event 39.0[8.2]) | Age, sex and education matched non-demented controls drawn from subjects relatives | 1.5T DTI | Age, sex, follow-up duration, education, lesion hemisphere, WMH volume, depressive symptoms, fatigue, stroke lesion volume, | 11 years after stroke WM FA positively related to and MD negatively related to an assessment of global cognitive function. Associations independent of WMH volume, stroke volume and other variables that were controlled for. WM integrity associated with processing speed, attention, working memory and executive function sub-domains. |
| Sedaghat et al. 2016(20) | 4294 | 63.6 (11.0) | Stroke free patients from population based cohort | None | 1.5T DTI | Age, sex, systolic blood pressure, diastolic blood pressure, antihypertensive medication, smoking, total cholesterol, HDL cholesterol, lipid lowering medication, diabetes, coronary heart disease, APOEe4 status, BMI, intracranial volume, WMH volume, WM volume, CMB, lacunar infarcts. | Each SD decrease in FA and increase in MD associated with hazard ratios for all cause mortality of 1.24(1.10-1.43) and 1.32(1.11-1.56) respectively. Looking at cardiovascular and non-cardiovascular mortality separately only significant risk after correction for other variables was FA decrease having a hazard ratio for cardiovascular mortality of 1.53 (1.04-2.24). |
| Van Uden et al. 2015(21) | 151 Depressive symptoms  287  Free from depressive symptoms | 65.6(8.6)  64.8 (8.9) | Depressive symptom subjects aged between 50 and 85 with WMH and or lacunar infarcts on neuroimaging | Depressive symptom free subjects aged between 50 and 85 with WMH and or lacunar infarcts on neuroimaging | 1.5T DTI | Age, sex, education level, normalised total brain volume, WMH volume, number of lacunar infarcts, MMSE score, cognitive index | Participants with depressive symptoms had lower FA and higher MD, axial diffusivity and radial diffusivity in the corpus callosum, inferior-fronto-occipital fasciculus, uncinate fasciculus and corona radiata. Association not affected by global cognitive function but there was no association after adjusting for WMH volume and lacunar infarcts. |
| Wang et al. 2015(22) | 241 | 72.0(9.0) | Age-stratified population random sample | None | 1.5T DTI | Age, sex, education, APOEe4, vascular risk factors, WMH volume, time to follow up | Decreased FA associated with heavy alcohol consumption whilst increased MD associated with hypertension and diabetes. FA decreased and MD increased with increasing number of vascular risk factors present in an individual. APOEe4 worsened the effect of vascular risk factors. Being in the lowest tertile of FA subjects and highest tertile of MD subjects was associated with the fastest decline in MMSE scores over six years follow up but unclear if this analysis adjusted for SVD features or risk factors. |
| Benjamin et al. 2016(23) | 121 | 70 (9.8) | Symptomatic lacunar stroke and WMH grade 2 on Fazekas scale | None | 1.5T DTI | None | Changes in brain volume, WMH volume, lacune number and MD detectable over three years but changes in processing speed and executive function not seen. Sample size estimates for use of imaging markers as outcome measure WMH volume change sample size < MD peak height change <brain volume change < lacune number change. |
| Kalheim et al. 2017(24) | 31 amyloid  20 controls | 63.8(6.9)  59.9(7.3) | MCI or subjective cognitive decline patients with abnormal CSF amyloid levels | MCI or subjective cognitive decline patients with normal CSF amyloid levels | 1.5T DTI | Age and WMH volume | MD, axial diffusivity and radial diffusivity higher in WMH of amyloid positive patients. No differences in FA. |
| Munoz-Maniega et al. 2016(25) | 204 | 66.0 (11.5) | Mild ischaemic stroke | None | 1.5T DTi and DCE-MRI | Age, Fazekas score, hypertension, smoking status and pulse pressure | Decreased FA, increased MD, increased water content and increased BBB leakage seen WMH compared to NAWM. Increasing WMH associated with deterioration in all these parameters in NAWM. Increasing age associated with increased MD and water content. |
| Tang et al. 2015(26) | 27 Subjects  30 Controls | 65.8±5.9  63.5±6.1 | Silent lacunar infarcts | Healthy controls | 3T DTI | Age, gender, brain volume | Global and local efficiencies were reduced in the lacunar infarct group compared to the controls. Memory, attention, executive function and verbal fluency were associated with nodal efficiency. |

References

1. Baykara E, Gesierich B, Adam R, Tuladhar AM, Biesbroek JM, Koek HL, et al. A Novel Imaging Marker for Small Vessel Disease Based on Skeletonization of White Matter Tracts and Diffusion Histograms. Annals of Neurology. 2016;80(4):581-92.

2. Chen Y, Wang A, Tang J, Wei D, Li P, Chen K, et al. Association of White Matter Integrity and Cognitive Functions in Patients with Subcortical Silent Lacunar Infarcts. Stroke. 2015;46(4):1123-6.

3. Evans TE, O'Sullivan MJ, De Groot M, Niessen WJ, Hofman A, Krestin GP, et al. White matter microstructure improves stroke risk prediction in the general population. Stroke. 2016;47(11):2756-62.

4. Hollocks MJ, Lawrence AJ, Brookes RL, Barrick TR, Morris RG, Husain M, et al. Differential relationships between apathy and depression with white matter microstructural changes and functional outcomes. Brain. 2015;138(12):3803-15.

5. Kim HJ, Im K, Kwon H, Lee JM, Kim C, Kim YJ, et al. Clinical effect of white matter network disruption related to amyloid and small vessel disease. Neurology. 2015;85(1):63-70.

6. Kim YJ, Kwon HK, Lee JM, Cho H, Kim HJ, Park HK, et al. Gray and white matter changes linking cerebral small vessel disease to gait disturbances. Neurology. 2016;86(13):1199-207.

7. Kim YJ, Kwon HK, Lee JM, Kim YJ, Kim HJ, Jung NY, et al. White matter microstructural changes in pure Alzheimer's disease and subcortical vascular dementia. Eur J Neurol. 2015;22:709-16.

8. Li Y, Wang D, Zhang H, Wang Y, Wu P, Zhang H, et al. Changes of Brain Connectivity in the Primary Motor Cortex After Subcortical Stroke: A Multimodal Magnetic Resonance Imaging Study. Medicine. 2016;95(6):e2579.

9. Liu J, Qin W, Zhang J, Zhang X, Yu C. Enhanced Interhemispheric Functional Connectivity Compensates for Anatomical Connection Damages in Subcortical Stroke. Stroke. 2015;46(4):1045-51.

10. Maillard P, Mitchell GF, Himali JJ, Beiser A, Tsao CW, Pase MP, et al. Effects of arterial stiffness on brain integrity in young adults from the framingham heart study. Stroke. 2016;47(4):1030-6.

11. McEvoy LK, Fennema-Notestine C, Eyler LT, Franz CE, Hagler DJ, Lyons MJ, et al. Hypertension-Related Alterations in White Matter Microstructure Detectable in Middle Age. Hypertension. 2015;66(2):317-23.

12. Mutlu U, Cremers LGM, De Groot M, Hofman A, Niessen WJ, Van Der Lugt A, et al. Retinal microvasculature and white matter microstructure. Neurology. 2016;87(10):1003-10.

13. Nadkarni NK, Boudreau RM, Studenski SA, Lopez OL, Liu G, Kritchevsky S, et al. Slow gait, white matter characteristics, and prior 10-year interleukin-6 levels in older adults. Neurology. 2016;87(19):1993-9.

14. Pasi M, Salvadori E, Poggesi A, Ciolli L, Del Bene A, Marini S, et al. White matter microstructural damage in small vessel disease is associated with montreal cognitive assessment but not with mini mental state examination performances: vascular mild cognitive impairment tuscany study. Stroke. 2015;46(1):262-4.

15. Reijmer YD, Fotiadis P, Martinez-Ramirez S, Salat DH, Schultz A, Shoamanesh A, et al. Structural network alterations and neurological dysfunction in cerebral amyloid angiopathy. Brain. 2015;138(1):179-88.

16. Sam K, Crawley AP, Conklin J, Poublanc J, Sobczyk O, Mandell DM, et al. Development of White Matter Hyperintensity Is Preceded by Reduced Cerebrovascular Reactivity. Annals of Neurology. 2016;80(2):277-85.

17. Sam K, Peltenburg B, Conklin J, Sobczyk O, Poublanc J, Crawley AP, et al. Cerebrovascular reactivity and white matter integrity. Neurology. 2016;87(22):2333-9.

18. Santiago C, Herrmann N, Swardfager W, Saleem M, Oh PI, Black SE, et al. White matter microstructural integrity is associated with executive function and processing speed in older adults with coronary artery disease. American Journal of Geriatric Psychiatry. 2015;23(7):754-63.

19. Schaapsmeerders P, Tuladhar AM, Arntz RM, Franssen S, Maaijwee NAM, Rutten-Jacobs LCA, et al. Remote Lower White Matter Integrity Increases the Risk of Long-Term Cognitive Impairment after Ischemic Stroke in Young Adults. Stroke. 2016;47(10):2517-25.

20. Sedaghat S, Cremers LGM, De Groot M, Hofman A, Van Der Lugt A, Niessen WJ, et al. Lower microstructural integrity of brain white matter is related to higher mortality. Neurology. 2016;87(9):927-34.

21. Van Uden IWM, Tuladhar AM, De Laat KF, Van Norden AGW, Norris DG, Van Dijk EJ, et al. White matter integrity and depressive symptoms in cerebral small vessel disease: The RUN DMC study. American Journal of Geriatric Psychiatry. 2015;23(5):525-35.

22. Wang R, Fratiglioni L, Laukka EJ, Lovden M, Kalpouzos G, Keller L, et al. Effects of vascular risk factors and APOE epsilon4 on white matter integrity and cognitive decline. Neurology. 2015;84(11):1128-35.

23. Benjamin P, Zeestraten E, Lambert C, Ster IC, Williams OA, Lawrence AJ, et al. Progression of MRI markers in cerebral small vessel disease: Sample size considerations for clinical trials. Journal of Cerebral Blood Flow & Metabolism. 2016;36(1):228-40.

24. Kalheim LF, Bjornerud A, Fladby T, Vegge K, Selnes P. White matter hyperintensity microstructure in amyloid dysmetabolism. J Cereb Blood Flow Metab. 2017;37(1):356-65.

25. Munoz Maniega S, Chappell FM, Valdes Hernandez MC, Armitage PA, Makin SD, Heye AK, et al. Integrity of normal-appearing white matter: influence of age, visible lesion burden and hypertension in patients with small vessel disease. J Cereb Blood Flow Metab. 2016;in press.

26. Tang J, Zhong S, Chen Y, Chen K, Zhang J, Gong G, et al. Aberrant white matter networks mediate cognitive impairment in patients with silent lacunar infarcts in basal ganglia territory. Journal of Cerebral Blood Flow & Metabolism. 2015;35(9):1426-34.

27. Bouvy WH, Geurts LJ, Kuijf HJ, Luijten PR, Kappelle LJ, Biessels GJ, et al. Assessment of blood flow velocity and pulsatility in cerebral perforating arteries with 7-T quantitative flow MRI. NMR Biomed. 2015;10.1002/nbm.3306.

28. Cooper LL, Woodard T, Sigurdsson S, van Buchem MA, Torjesen AA, Inker LA, et al. Cerebrovascular Damage Mediates Relations Between Aortic Stiffness and Memory. Hypertension. 2016;67(1):176-82.

29. Doi H, Inamizu S, Saito BY, Murai H, Araki T, Kira JI. Analysis of cerebral lobar microbleeds and a decreased cerebral blood flow in a memory clinic setting. Internal Medicine. 2015;54(9):1027-33.

30. Foster-Dingley JC, Moonen JE, de Craen AJ, de Ruijter W, van der Mast RC, van der Grond J. Blood Pressure Is Not Associated With Cerebral Blood Flow in Older Persons. Hypertension. 2015;66(5):954-60.

31. Hoscheidt SM, Kellawan JM, Berman SE, Rivera-Rivera LA, Krause RA, Oh JM, et al. Insulin resistance is associated with lower arterial blood flow and reduced cortical perfusion in cognitively asymptomatic middle-aged adults. J Cereb Blood Flow Metab. 2016.

32. Nasel C, Boubela R, Kalcher K, Moser E. Normalised time-to-peak-distribution curves correlate with cerebral white matter hyperintensities - Could this improve early diagnosis? J Cereb Blood Flow Metab. 2016.

33. Promjunyakul NO, Lahna DL, Kaye JA, Dodge HH, Erten-Lyons D, Rooney WD, et al. Comparison of cerebral blood flow and structural penumbras in relation to white matter hyperintensities: A multi-modal magnetic resonance imaging study. J Cereb Blood Flow Metab. 2016;36(9):1528-36.

34. Promjunyakul N, Lahna D, Kaye JA, Dodge HH, Erten-Lyons D, Rooney WD, et al. Characterizing the white matter hyperintensity penumbra with cerebral blood flow measures. NeuroImage: Clinical. 2015;8:224-9.

35. Al-Bachari S, Vidyasagar R, Emsley HC, Parkes LM. Structural and physiological neurovascular changes in idiopathic Parkinson's disease and its clinical phenotypes. J Cereb Blood Flow Metab. 2017:271678x16688919.

36. Zarrinkoob L, Ambarki K, Wahlin A, Birgander R, Carlberg B, Eklund A, et al. Aging alters the dampening of pulsatile blood flow in cerebral arteries. J Cereb Blood Flow Metab. 2016;36(9):1519-27.

37. Zonneveld HI, Loehrer EA, Hofman A, Niessen WJ, van der Lugt A, Krestin GP, et al. The bidirectional association between reduced cerebral blood flow and brain atrophy in the general population. Journal of Cerebral Blood Flow & Metabolism. 2015;35(11):1882-7.

38. Huisa BN, Caprihan A, Thompson J, Prestopnik J, Qualls CR, Rosenberg GA. Long-term blood-brain barrier permeability changes in Binswanger disease. Stroke. 2015;46(9):2413-8.

39. Yang J, d'Esterre C, Ceruti S, Roversi G, Saletti A, Fainardi E, et al. Temporal changes in blood-brain barrier permeability and cerebral perfusion in lacunar/subcortical ischemic stroke. BMC Neurology. 2015;15 (1) (no pagination)(214).

40. Heye AK, Thrippleton MJ, Armitage PA, Valdes Hernandez MC, Makin SD, Glatz A, et al. Tracer kinetic modelling for DCE-MRI quantification of subtle blood-brain barrier permeability. Neuroimage. 2016;125:446-55.

41. van de Haar HJ, Burgmans S, Jansen JF, van Osch MJ, van Buchem MA, Muller M, et al. Blood-brain barrier leakage in patients with early Alzheimer disease. Radiology. 2016;281(2):527-35.

42. Wardlaw JM, Makin SJ, Valdés Hernández MC, Armitage PA, Heye AK, Chappell FM, et al. Blood-brain barrier failure as a core mechanism in cerebral small vessel disease and dementia: evidence from a cohort study. Alzheimer's & Dementia: The Journal of the Alzheimer's Association.

43. Montagne A, Barnes SR, Sweeney MD, Halliday MR, Sagare AP, Zhao Z, et al. Blood-brain barrier breakdown in the aging human hippocampus. Neuron. 2015;85(2):296-302.
