# Supplementary material for: Advanced Neuroimaging of Cerebral Small Vessel Disease
Source: Curr Treat Options Cardiovasc Med. 2017 Jun 15;19(7):56. doi: 10.1007/s11936-017-0555-1 (PMC5486578; doi:10.1007/s11936-017-0555-1)
Supplement: Supplementary file 3 — (DOC 41 kb). [file 11936_2017_555_MOESM3_ESM.doc]

**Search Strategies**

**SVD Score 22/12/2016**

Database: Embase <1980 to 2016 Week 51>, Ovid MEDLINE(R) In-Process & Other Non-Indexed Citations and Ovid MEDLINE(R) <1946 to Present>

Search Strategy:

--------------------------------------------------------------------------------

1 brain isch?emia/ or brain infarction/ or cerebral infarction/ or hypoxia-isch?emia, brain/ or stroke$.tw. (624424)

2 cerebral vascular attack/ or cerebrovascular attack/ or cerebral vascular accident/ or cerebrovascular accident/ or CVA.tw. (255923)

3 (lacun$ or small vessel$ or small infarct$ or microinfarct$ or subcortical lesion$ or subcortical infarct$).tw. (54637)

4 leukoaraiosis.tw. (2372)

5 Vascular dementia$/ or multi-infarct dementia$/ or subcortical dementia$.tw. (17953)

6 white matter hyperintensit$/ or WMH.tw. (3232)

7 small vessel disease/ or SVD.tw. (4317)

8 white matter lesion$/ or WML.tw. (5743)

9 (microbleed$ or microh?emorrhage$).mp. [mp=ti, ab, hw, tn, ot, dm, mf, dv, kw, fs, nm, kf, px, rx, ui] (5529)

10 (perivascular space$ or PVS or enlarged perivascular space$ or EPVS).mp. [mp=ti, ab, hw, tn, ot, dm, mf, dv, kw, fs, nm, kf, px, rx, ui] (12136)

11 1 or 2 or 3 or 4 or 5 or 6 or 7 or 8 or 9 or 10 (754858)

12 Small vessel disease score$/ or SVD score$.tw. (35)

13 Small vessel disease burden$/ or SVD burden$.tw. (25)

14 Small vessel disease quantification$/ or SVD quantification$.tw. (1)

15 12 or 13 (58)

16 remove duplicates from 15 (32)

17 limit 16 to yr="2015 -Current" (24)

**SVD Burden search 2 22/12/2016**

Database: Embase <1980 to 2016 Week 51>, Ovid MEDLINE(R) In-Process & Other Non-Indexed Citations and Ovid MEDLINE(R) <1946 to Present>

Search Strategy:

--------------------------------------------------------------------------------

1 brain isch?emia/ or brain infarction/ or cerebral infarction/ or hypoxia-isch?emia, brain/ or stroke$.tw. (624424)

2 cerebral vascular attack/ or cerebrovascular attack/ or cerebral vascular accident/ or cerebrovascular accident/ or CVA.tw. (255923)

3 (lacun$ or small vessel$ or small infarct$ or microinfarct$ or subcortical lesion$ or subcortical infarct$).tw. (54637)

4 leukoaraiosis.tw. (2372)

5 Vascular dementia$/ or multi-infarct dementia$/ or subcortical dementia$.tw. (17953)

6 white matter hyperintensit$/ or WMH.tw. (3232)

7 small vessel disease/ or SVD.tw. (4317)

8 white matter lesion$/ or WML.tw. (5743)

9 (microbleed$ or microh?emorrhage$).mp. [mp=ti, ab, hw, tn, ot, dm, mf, dv, kw, fs, nm, kf, px, rx, ui] (5529)

10 (perivascular space$ or PVS or enlarged perivascular space$ or EPVS).mp. [mp=ti, ab, hw, tn, ot, dm, mf, dv, kw, fs, nm, kf, px, rx, ui] (12136)

11 1 or 2 or 3 or 4 or 5 or 6 or 7 or 8 or 9 or 10 (754858)

12 Small vessel disease score$/ or SVD score$.tw. (35)

13 Small vessel disease burden$/ or SVD burden$.tw. (25)

14 Small vessel disease quantification$/ or SVD quantification$.tw. (1)

15 12 or 13 (58)

16 remove duplicates from 15 (32)

17 limit 16 to yr="2015 -Current" (24)

18 quantification.tw. (285196)

19 score.tw. (1037352)

20 burden.tw. (335504)

21 magnetic resonance imaging/ or MRI.tw. (946272)

22 18 or 19 or 20 (1626822)

23 11 and 21 and 22 (7394)

24 limit 23 to full text (2578)

25 limit 24 to yr="2015 -Current" (488)

**Vascular malfunction search 22/12/2016**

Database: Embase <1980 to 2016 Week 51>, Ovid MEDLINE(R) In-Process & Other Non-Indexed Citations and Ovid MEDLINE(R) <1946 to Present>

Search Strategy:

--------------------------------------------------------------------------------

1 brain isch?emia/ or brain infarction/ or cerebral infarction/ or hypoxia-isch?emia, brain/ or stroke$.tw. (624424)

2 cerebral vascular attack/ or cerebrovascular attack/ or cerebral vascular accident/ or cerebrovascular accident/ or CVA.tw. (255923)

3 (lacun$ or small vessel$ or small infarct$ or microinfarct$ or subcortical lesion$ or subcortical infarct$).tw. (54637)

4 leukoaraiosis.tw. (2372)

5 Vascular dementia$/ or multi-infarct dementia$/ or subcortical dementia$.tw. (17953)

6 white matter hyperintensit$/ or WMH.tw. (3232)

7 small vessel disease/ or SVD.tw. (4317)

8 white matter lesion$/ or WML.tw. (5743)

9 (microbleed$ or microh?emorrhage$).mp. [mp=ti, ab, hw, tn, ot, dm, mf, dv, kw, fs, nm, kf, px, rx, ui] (5529)

10 (perivascular space$ or PVS or enlarged perivascular space$ or EPVS).mp. [mp=ti, ab, hw, tn, ot, dm, mf, dv, kw, fs, nm, kf, px, rx, ui] (12136)

11 1 or 2 or 3 or 4 or 5 or 6 or 7 or 8 or 9 or 10 (754858)

12 cerebral vascular reactivity/ or cerebrovascular reactivity/ or cerebral vasoreactivity/ or CVR.tw. (4509)

13 cerebral blood flow/ or CBF.tw. (121707)

14 cerebral autoregulation.tw. (3899)

15 blood brain barrier permeability/ or BBB permeability.tw. (4754)

16 12 or 13 or 14 or 15 (130437)

17 magnetic resonance imaging/ or MRI.tw. (946272)

18 functional.tw. (2280185)

19 fMRI.tw. (78516)

20 blood oxygen level dependent/ or BOLD.tw. (25655)

21 arterial spin labelling/ or ASL.tw. (8219)

22 dynamic contrast enhanced/ or DCE.tw. (10400)

23 17 or 18 or 19 or 20 or 21 or 22 (3145889)

24 11 and 16 and 23 (7410)

25 limit 24 to full text (2207)

26 limit 25 to yr="2015 -Current" (241)

27 remove duplicates from 26 (177)

***************************

**White Matter Integrity Search**

Database: Ovid MEDLINE(R) In-Process & Other Non-Indexed Citations and Ovid MEDLINE(R) <1946 to Present>, Embase <1974 to 2017 January 11>, Ovid MEDLINE(R) Epub Ahead of Print <January 11, 2017>

Search Strategy:

--------------------------------------------------------------------------------

1 (A Novel Imaging Marker for Small Vessel Disease Based on Skeletonization of White Matter Tracts and Diffusion Histograms).mp. [mp=ti, ab, ot, nm, hw, kf, px, rx, ui, tn, dm, mf, dv, kw, fs] (2)

2 limit 1 to full text (2)

3 limit 2 to yr="2015 -Current" (2)

4 brain isch?emia/ or brain infarction/ or cerebral infarction/ or hypoxia-isch?emia, brain/ or stroke$.mp. (732304)

5 cerebral vascular attack/ or cerebrovascular attack/ or cerebral vascular accident/ or cerebrovascular accident/ or CVA.mp. (261259)

6 (lacun$ or small vessel$ or small infarct$ or microinfarct$ or subcortical lesion$ or subcortical infarct$).mp. (57834)

7 leukoaraiosis.mp. (3282)

8 Vascular dementia$/ or multi-infarct dementia$/ or subcortical dementia$.mp. (18095)

9 white matter hyperintensit$/ or WMH.mp. (3337)

10 small vessel disease/ or SVD.mp. (4567)

11 white matter lesion$/ or WML.mp. (5845)

12 (perivascular space$ or PVS or enlarged perivascular space$ or EPVS).mp. (12498)

13 4 or 5 or 6 or 7 or 8 or 9 or 10 or 11 or 12 (846338)

14 Mean diffusivity/ or MD.mp. (124599)

15 Fractional anisotropy/ or FA.mp. (72416)

16 tractography.mp. (10982)

17 integrity.mp. (251530)

18 microstructur$.mp. (59958)

19 microstructur$.mp. (59958)

20 diffus$ tensor imag$.mp. (35506)

21 dti.mp. (22324)

22 14 or 15 or 16 or 17 or 18 or 19 or 20 or 21 (512586)

23 13 and 22 (11769)

24 limit 23 to full text (2948)

25 limit 24 to yr="2015 -Current" (665)

26 remove duplicates from 25 (429)

***************************
