# Supplementary material for: Advanced Neuroimaging of Cerebral Small Vessel Disease
Source: Curr Treat Options Cardiovasc Med. 2017 Jun 15;19(7):56. doi: 10.1007/s11936-017-0555-1 (PMC5486578; doi:10.1007/s11936-017-0555-1)
Supplement: Supplementary file 4 — (DOC 207 kb). [file 11936_2017_555_MOESM4_ESM.doc]

**Table S3**: Original papers published since 1^st^ January 2015 on vascular malfunction and SVD

| Study | N | Age | SVD Group | Control Group | Imaging Method | Variables Controlled For | Main Findings |
| --- | --- | --- | --- | --- | --- | --- | --- |
| **Cerebral Blood Flow** | | | | | | | |
| Bouvy et al 2016(27) | 6 | 23-29 | N/A | Young healthy controls | 7T PC-MRI | N/A | Measured blood flow velocity and pulsatility in individual perforating arteries down to 80 micrometres |
| Cooper et al 2016(28) | 1820 | 80±5 | Population based healthy elderly cohort | N/A | 1.5T PC-MRI | Age, height, weight, heart rate,  diabetes mellitus, previous cardiovascular disease, use of antihyper-  tensive and lipid-lowering medication, total and high-density lipopro-  tein cholesterol levels, triglycerides, smoking, education level, and  depressive symptoms. | Increased carotid femoral PWV associated with decreased memory score. Cerebrovascular resistance (mean arterial pressure /CVF) and WMH both associated with PWV and memory and both attenuated the direct relationship between PWV and memory. |
| Doi(29) | 36 patients  86 controls | 81.3±5.4  77.7±8.4 | Patients with lobar cerebral microbleeds from a mixed cohort of Alzheimer’s disease, vascular dementia, mixed dementia, Lewy-body dementia, mild cognitive impairment and subjective cognitive  impairment. | Patients without lobar cerebral microbleeds from a mixed cohort of Alzheimer’s disease, vascular dementia, mixed dementia, Lewy-body dementia, mild cognitive impairment and subjective cognitive impairment. | 1.5T SWAN MRI for microbleeds  99Tc-ECD SPECT for CBF (qualitative measure) | None stated | 17 out of 23 patients with microbleeds had decreased CBF within 1cm of the microbleed on SPECT imaging. Parietal and occipital microbleeds were more associated with decreased CBF than frontal microbleeds and when comparing microbleed burdens areas with five or more microbleeds had reduced CBF compared to areas with just one |
| Foster-Dingley et al. 2015 (30) | 203 cross-sectional analysis  102 longitudinal analysis | 80.8(4.1)  80.5(3.9) | Cross sectional:  Stroke free community dwelling subjects on antihypertensive medication with an MMSE score of 21-27  Longitudinal: as above – discontinued antihypertensive therapy | Cross-sectional: None  Longitudinal study:  Subjects who continue antihypertensive therapy | 3T pcASL | Age and sex | Cross-sectional:  Found no association between CBF and any blood pressure parameter (systolic BP, diastolic BP, pulse pressue, mean arterial pressure or change on standing in systolic or diastolic BP). This included an analysis of subgroups with WMH volume, presence of microbleeds, and presence of lacunar infarcts.  Longitudinal:  No difference in change in CBF between group who discontinued and continued antihypertensive treatment. |
| Hoscheidt et al 2016 (31) | 120 | 57±5 | Asymptomatic subjects but cohort enriched with 78% having a parental history of Alzheimer’s disease. Exact inclusion criteria not specified | None | 3T pcASL and PC-MRI | Age, sex, waist circumference, MAP,triglycerides, HDL and total cholesterol, APOEe4 carriage, grey matter volume | Insulin resistance was associated with reduced arterial flow on phase contrast MRI and reduced cerebral perfusion on ASL in a number of grey matter regions |
| Nasel et al. 2016 (32) | 50 SVD patients  38 elderly controls  18 young controls | SVD:  75.7±9.6  Elderly controls:  68.4±9.0  Young controls:  42.3±8.5 | Random sample from institutional database of >6000 scans. Groups decided based on age and WMH volume.  SVD group considered to be those aged >53 with WMH volume greater than 2x the median absolute deviation from the regression line of the “young control” group WMH volume.  Mean 52.1±7.7cm3 WMH | Young controls:  Age <53 (note this group had mena WMH volume of 10.4±2.3cm3)  Elderly controls:  Age ≥ 53 with WMH below the threshold of 2x median absolute deviation from the regression line. (Mean WMH volume 17.1±5.5) | 1.5T DCE MRI | Age, hypertension gender, diabetes, hyperlipidaemia and smoking. Although notes the retrospective design meant vascular risk factors could not be specifically assessed. | Increased arteriovenous transit time was seen in patients with SVD compared to both elderly and young controls. Decreased bolus spread velocity was seen in those with an increased WMH volume.  Age was a strong confounder however and hypertension also had a weak confounding effect. Gender, diabetes, hyperlipidaemia and smoking did not affect the results. |
| Promjunyakul et al 2016 (33) | 82 | 84(8.2) | Cognitively intact healthy elderly with WMH | None | 3T ASL and DTI | None | Reduced CBF around a WMH extended to 13-14mm around the WMH. Microstructural abnormalities measured by FA/MD and FLAIR intensity extended to 2-9mm around WMH. |
| Promjunyakul et al 2015 (34) | Cross-sectional;  61  Longitudinal:  24 | 84.6(8.0) | Cognitively intact healthy elderly with WMH | None | 3T ASL | None | CBF reduced compared to other NAWM out to a distance of 12mm from WMH and in 24 who returned for follow up areas developing new WMH after 18 months had lower CBF on the baseline scan |
| Al-Bachari et al 2017 (35) | 18 Cerebrovascular Disease  51 Idiopathic Parkinson’s disease  34 Controls | 70.1 (53-84)  69.0(52-85)  67.4(52-85) | Cerebrovascular disease patients (not specified in more detail). Median WMH volume 10.44ml  PD patients. Median WMH volume 4ml | Free of cerebrovascular disease and PD. Median WMH volume 1.41ml | 3T ASL (note 14 controls and 14 PD patients were scanned with an 8 channel head coil rather than the 32 channel used for all other participants) | Gender, PD disease severity and duration. | Patients with stroke and Parkinson’s disease were seen to have a prolonged arterial arrival time compared to healthy age matched controls. Both the stroke group and the PD group had similar WMH burdens.  No association of AAT with MoCA score. |
| Zarrinkoob et al. (36) | 45 subjects  49 controls | 71±4  25±2 | Healthy elderly | Healthy young | PC-MRI | Age and sex corrected for when analysing correlation of PWV with PI. | Pulsatility index in older subjects higher compared to younger subjects and this was more pronounced in distal arteries. Dampening of the pulsatility index across the cerebral arteries was also reduced in the elderly cohort. PWV not correlated with PI. |
| Zonneveld et al. 2015 (37) | 3011 | 59.6(8.0) | Non demented subjects from a population based cohort. | None | PC-MRI | Age, sex, scan interval, intracranial volume, GM volume, WM volume, APOEe4 status, systolic and diastolic blood pressure, antihypertensive medication, BMI, diabetes, total and HDL cholesterol, lipid lowering medication, smoking, presence of carotid stenosis. | Lower brain volume at baseline was associated with reduced CBF at follow up. Decreased CBF was only associated with decreased brain volume in participants over 65. The analysis used follow up CBF as the dependent variable while including baseline CBF as an independent variable in a multivariable model, rather than looking directly at CBF change |
| **Cerebrovascular Reactivity** | | | | | | | |
| Sam et al 2016(16) | 45 | 74 (SD 9.4) | Patients >50 yrs presenting to neurology clinic with a range of symptoms with MRI showing WMH ≥2 on Fazekas scale | None | 3T BOLD and DTI MRI | None | CVR and FA decreased, whilst MD and T2 values increased in NAWM that progressed to WMH compared to WM in contralateral hemisphere that did not progress to WMH. |
| Sam et al 2016(17) | 75 | 74( SD 9.7) | Patients >50 yrs presenting to neurology clinic with a range of symptoms with MRI showing WMH ≥2 on Fazekas scale | None | 3T BOLD and DTI MRI | None | Negative CVR (suggestive of a vascular steal phenomenon) associated with decreased FA, CBF, cerebral blood volume and increased MD and time to maximum compared to regions with positive CVR |
| **BBB Leakage** | | | | | | | |
| Huisa et al. 2015 (38) | 22 patients  16 Controls | 67±10  61±9.5 | Subjects chosen retrospectively from a larger vascular cognitive impairment cohort. Presence of SVD defined as large WMHs (Fazekas score>2[not stated f deep or periventricular or combned]),>2 vascular risk factors and focal neurological symptoms or gait disturbance | Age matched controls. Not stated if from vascular cognitive impairment cohort or if healthy controls | DCE-MRI (TAPIR sequence)  14 subjects at 1.5T  8 subjects at 3T  Not stated what strength used for controls | None | BBB permeability seen to be increased and to have greater variability in terms of the areas it affected in patients with SVD compared to controls. Interestingly very little of the permeability was seen in the WMH themselves with most of the permeability confined to a 4mm ring around existing WMH |
| Yang et al. 2015(39) | 14 Patients  17 Controls | 71±10  69±12 | Lacunar infarcts | Cortical infarcts | CT perfusion | None | Increased BBB permeability in contralateral, non-infarcted basal ganglia/thalamus over first three months after stroke. |
| Heye et al 2016(40) | 201 | 66±11.5 | Mild stroke patients | None | 1.5T DCE-MRI | Scanner drift | BBB leakage (Ktrans and Vp) significantly increased in WMH compared to NAWM |
| Van de Haar et al 2016 (41) | 16 patients  17 controls | 73.6±7.9  75.8±6.2 | Alzheimer’s disease with a moderate volume of WMH (mean 15.8ml) | Healthy controls | 3T DCE-MRI | Age, sex, WMH volume, diabetes and other non-cerebral vascular disease | In patients BBB leakage rate was increased in total grey matter and cortex, whilst volume fraction of leakage tissue was increased in total grey matter, NAWM, deep grey matter and cortex. MMSE scores decreased with increasing leakage volume in deep GM and cortex. |
| Wardlaw et al (42) same cohort as Heye, ref 64 | 201 | 67 (34-97) | Mild ischaemic stroke patients | None | 1.5T DCE-MRI | Age, WMH burden, vascular risk factors, intravascular contrast baseline T1 value and time after contrast injection. | BBB leakage and interstitial fluid increased in WMH compared to NAWM. Leakage increased in NAWM with proximity to WMH. Leakage increased with WMH severity, age and hypertension. BBB leakage predicted declining cognition at one year. |
| Montagne et al. 2015(43) | MCI 21  Older controls 18  Young controls 6  Multiple sclerosis  19 | 55-85  55-91  23-47  26-53 | Mild cognitive impairment patients | Not stated how controls were recruited or if had vascular risk factors | 3T DCE-MRI | None | BBB leakage in the hippocampus increased with age and was further increased by the presence of mild cognitive impairment. |

References

1. Baykara E, Gesierich B, Adam R, Tuladhar AM, Biesbroek JM, Koek HL, et al. A Novel Imaging Marker for Small Vessel Disease Based on Skeletonization of White Matter Tracts and Diffusion Histograms. Annals of Neurology. 2016;80(4):581-92.

2. Chen Y, Wang A, Tang J, Wei D, Li P, Chen K, et al. Association of White Matter Integrity and Cognitive Functions in Patients with Subcortical Silent Lacunar Infarcts. Stroke. 2015;46(4):1123-6.

3. Evans TE, O'Sullivan MJ, De Groot M, Niessen WJ, Hofman A, Krestin GP, et al. White matter microstructure improves stroke risk prediction in the general population. Stroke. 2016;47(11):2756-62.

4. Hollocks MJ, Lawrence AJ, Brookes RL, Barrick TR, Morris RG, Husain M, et al. Differential relationships between apathy and depression with white matter microstructural changes and functional outcomes. Brain. 2015;138(12):3803-15.

5. Kim HJ, Im K, Kwon H, Lee JM, Kim C, Kim YJ, et al. Clinical effect of white matter network disruption related to amyloid and small vessel disease. Neurology. 2015;85(1):63-70.

6. Kim YJ, Kwon HK, Lee JM, Cho H, Kim HJ, Park HK, et al. Gray and white matter changes linking cerebral small vessel disease to gait disturbances. Neurology. 2016;86(13):1199-207.

7. Kim YJ, Kwon HK, Lee JM, Kim YJ, Kim HJ, Jung NY, et al. White matter microstructural changes in pure Alzheimer's disease and subcortical vascular dementia. Eur J Neurol. 2015;22:709-16.

8. Li Y, Wang D, Zhang H, Wang Y, Wu P, Zhang H, et al. Changes of Brain Connectivity in the Primary Motor Cortex After Subcortical Stroke: A Multimodal Magnetic Resonance Imaging Study. Medicine. 2016;95(6):e2579.

9. Liu J, Qin W, Zhang J, Zhang X, Yu C. Enhanced Interhemispheric Functional Connectivity Compensates for Anatomical Connection Damages in Subcortical Stroke. Stroke. 2015;46(4):1045-51.

10. Maillard P, Mitchell GF, Himali JJ, Beiser A, Tsao CW, Pase MP, et al. Effects of arterial stiffness on brain integrity in young adults from the framingham heart study. Stroke. 2016;47(4):1030-6.

11. McEvoy LK, Fennema-Notestine C, Eyler LT, Franz CE, Hagler DJ, Lyons MJ, et al. Hypertension-Related Alterations in White Matter Microstructure Detectable in Middle Age. Hypertension. 2015;66(2):317-23.

12. Mutlu U, Cremers LGM, De Groot M, Hofman A, Niessen WJ, Van Der Lugt A, et al. Retinal microvasculature and white matter microstructure. Neurology. 2016;87(10):1003-10.

13. Nadkarni NK, Boudreau RM, Studenski SA, Lopez OL, Liu G, Kritchevsky S, et al. Slow gait, white matter characteristics, and prior 10-year interleukin-6 levels in older adults. Neurology. 2016;87(19):1993-9.

14. Pasi M, Salvadori E, Poggesi A, Ciolli L, Del Bene A, Marini S, et al. White matter microstructural damage in small vessel disease is associated with montreal cognitive assessment but not with mini mental state examination performances: vascular mild cognitive impairment tuscany study. Stroke. 2015;46(1):262-4.

15. Reijmer YD, Fotiadis P, Martinez-Ramirez S, Salat DH, Schultz A, Shoamanesh A, et al. Structural network alterations and neurological dysfunction in cerebral amyloid angiopathy. Brain. 2015;138(1):179-88.

16. Sam K, Crawley AP, Conklin J, Poublanc J, Sobczyk O, Mandell DM, et al. Development of White Matter Hyperintensity Is Preceded by Reduced Cerebrovascular Reactivity. Annals of Neurology. 2016;80(2):277-85.

17. Sam K, Peltenburg B, Conklin J, Sobczyk O, Poublanc J, Crawley AP, et al. Cerebrovascular reactivity and white matter integrity. Neurology. 2016;87(22):2333-9.

18. Santiago C, Herrmann N, Swardfager W, Saleem M, Oh PI, Black SE, et al. White matter microstructural integrity is associated with executive function and processing speed in older adults with coronary artery disease. American Journal of Geriatric Psychiatry. 2015;23(7):754-63.

19. Schaapsmeerders P, Tuladhar AM, Arntz RM, Franssen S, Maaijwee NAM, Rutten-Jacobs LCA, et al. Remote Lower White Matter Integrity Increases the Risk of Long-Term Cognitive Impairment after Ischemic Stroke in Young Adults. Stroke. 2016;47(10):2517-25.

20. Sedaghat S, Cremers LGM, De Groot M, Hofman A, Van Der Lugt A, Niessen WJ, et al. Lower microstructural integrity of brain white matter is related to higher mortality. Neurology. 2016;87(9):927-34.

21. Van Uden IWM, Tuladhar AM, De Laat KF, Van Norden AGW, Norris DG, Van Dijk EJ, et al. White matter integrity and depressive symptoms in cerebral small vessel disease: The RUN DMC study. American Journal of Geriatric Psychiatry. 2015;23(5):525-35.

22. Wang R, Fratiglioni L, Laukka EJ, Lovden M, Kalpouzos G, Keller L, et al. Effects of vascular risk factors and APOE epsilon4 on white matter integrity and cognitive decline. Neurology. 2015;84(11):1128-35.

23. Benjamin P, Zeestraten E, Lambert C, Ster IC, Williams OA, Lawrence AJ, et al. Progression of MRI markers in cerebral small vessel disease: Sample size considerations for clinical trials. Journal of Cerebral Blood Flow & Metabolism. 2016;36(1):228-40.

24. Kalheim LF, Bjornerud A, Fladby T, Vegge K, Selnes P. White matter hyperintensity microstructure in amyloid dysmetabolism. J Cereb Blood Flow Metab. 2017;37(1):356-65.

25. Munoz Maniega S, Chappell FM, Valdes Hernandez MC, Armitage PA, Makin SD, Heye AK, et al. Integrity of normal-appearing white matter: influence of age, visible lesion burden and hypertension in patients with small vessel disease. J Cereb Blood Flow Metab. 2016;in press.

26. Tang J, Zhong S, Chen Y, Chen K, Zhang J, Gong G, et al. Aberrant white matter networks mediate cognitive impairment in patients with silent lacunar infarcts in basal ganglia territory. Journal of Cerebral Blood Flow & Metabolism. 2015;35(9):1426-34.

27. Bouvy WH, Geurts LJ, Kuijf HJ, Luijten PR, Kappelle LJ, Biessels GJ, et al. Assessment of blood flow velocity and pulsatility in cerebral perforating arteries with 7-T quantitative flow MRI. NMR Biomed. 2015;10.1002/nbm.3306.

28. Cooper LL, Woodard T, Sigurdsson S, van Buchem MA, Torjesen AA, Inker LA, et al. Cerebrovascular Damage Mediates Relations Between Aortic Stiffness and Memory. Hypertension. 2016;67(1):176-82.

29. Doi H, Inamizu S, Saito BY, Murai H, Araki T, Kira JI. Analysis of cerebral lobar microbleeds and a decreased cerebral blood flow in a memory clinic setting. Internal Medicine. 2015;54(9):1027-33.

30. Foster-Dingley JC, Moonen JE, de Craen AJ, de Ruijter W, van der Mast RC, van der Grond J. Blood Pressure Is Not Associated With Cerebral Blood Flow in Older Persons. Hypertension. 2015;66(5):954-60.

31. Hoscheidt SM, Kellawan JM, Berman SE, Rivera-Rivera LA, Krause RA, Oh JM, et al. Insulin resistance is associated with lower arterial blood flow and reduced cortical perfusion in cognitively asymptomatic middle-aged adults. J Cereb Blood Flow Metab. 2016.

32. Nasel C, Boubela R, Kalcher K, Moser E. Normalised time-to-peak-distribution curves correlate with cerebral white matter hyperintensities - Could this improve early diagnosis? J Cereb Blood Flow Metab. 2016.

33. Promjunyakul NO, Lahna DL, Kaye JA, Dodge HH, Erten-Lyons D, Rooney WD, et al. Comparison of cerebral blood flow and structural penumbras in relation to white matter hyperintensities: A multi-modal magnetic resonance imaging study. J Cereb Blood Flow Metab. 2016;36(9):1528-36.

34. Promjunyakul N, Lahna D, Kaye JA, Dodge HH, Erten-Lyons D, Rooney WD, et al. Characterizing the white matter hyperintensity penumbra with cerebral blood flow measures. NeuroImage: Clinical. 2015;8:224-9.

35. Al-Bachari S, Vidyasagar R, Emsley HC, Parkes LM. Structural and physiological neurovascular changes in idiopathic Parkinson's disease and its clinical phenotypes. J Cereb Blood Flow Metab. 2017:271678x16688919.

36. Zarrinkoob L, Ambarki K, Wahlin A, Birgander R, Carlberg B, Eklund A, et al. Aging alters the dampening of pulsatile blood flow in cerebral arteries. J Cereb Blood Flow Metab. 2016;36(9):1519-27.

37. Zonneveld HI, Loehrer EA, Hofman A, Niessen WJ, van der Lugt A, Krestin GP, et al. The bidirectional association between reduced cerebral blood flow and brain atrophy in the general population. Journal of Cerebral Blood Flow & Metabolism. 2015;35(11):1882-7.

38. Huisa BN, Caprihan A, Thompson J, Prestopnik J, Qualls CR, Rosenberg GA. Long-term blood-brain barrier permeability changes in Binswanger disease. Stroke. 2015;46(9):2413-8.

39. Yang J, d'Esterre C, Ceruti S, Roversi G, Saletti A, Fainardi E, et al. Temporal changes in blood-brain barrier permeability and cerebral perfusion in lacunar/subcortical ischemic stroke. BMC Neurology. 2015;15 (1) (no pagination)(214).

40. Heye AK, Thrippleton MJ, Armitage PA, Valdes Hernandez MC, Makin SD, Glatz A, et al. Tracer kinetic modelling for DCE-MRI quantification of subtle blood-brain barrier permeability. Neuroimage. 2016;125:446-55.

41. van de Haar HJ, Burgmans S, Jansen JF, van Osch MJ, van Buchem MA, Muller M, et al. Blood-brain barrier leakage in patients with early Alzheimer disease. Radiology. 2016;281(2):527-35.

42. Wardlaw JM, Makin SJ, Valdés Hernández MC, Armitage PA, Heye AK, Chappell FM, et al. Blood-brain barrier failure as a core mechanism in cerebral small vessel disease and dementia: evidence from a cohort study. Alzheimer's & Dementia: The Journal of the Alzheimer's Association.

43. Montagne A, Barnes SR, Sweeney MD, Halliday MR, Sagare AP, Zhao Z, et al. Blood-brain barrier breakdown in the aging human hippocampus. Neuron. 2015;85(2):296-302.
